# Germ Granule Evolution Provides Mechanistic Insight into *Drosophila* Germline Development

**DOI:** 10.1093/molbev/msad174

**Published:** 2023-08-01

**Authors:** Dominique A Doyle, Florencia N Burian, Benjamin Aharoni, Annabelle J Klinder, Melissa M Menzel, Gerard Carlo C Nifras, Ahad L Shabazz-Henry, Bianca Ulrich Palma, Gisselle A Hidalgo, Christopher J Sottolano, Bianca M Ortega, Matthew G Niepielko

**Affiliations:** School of Integrative Science and Technology, Kean University, Union, NJ, USA; School of Integrative Science and Technology, Kean University, Union, NJ, USA; School of Integrative Science and Technology, Kean University, Union, NJ, USA; School of Integrative Science and Technology, Kean University, Union, NJ, USA; School of Integrative Science and Technology, Kean University, Union, NJ, USA; School of Integrative Science and Technology, Kean University, Union, NJ, USA; School of Integrative Science and Technology, Kean University, Union, NJ, USA; School of Integrative Science and Technology, Kean University, Union, NJ, USA; School of Integrative Science and Technology, Kean University, Union, NJ, USA; Center for Computational and Integrative Biology, Rutgers, The State University of New Jersey, Camden, NJ, USA; School of Integrative Science and Technology, Kean University, Union, NJ, USA; School of Integrative Science and Technology, Kean University, Union, NJ, USA; Department of Biological Sciences, Kean University, Union, NJ, USA

**Keywords:** germ granules, polar granules, primordial germ cells, pole cells, germplasm, ribonucleoproteins, evolution, biomolecular condensates, homotypic clusters, *Drosophila*, mRNA localization, *nanos*

## Abstract

The copackaging of mRNAs into biomolecular condensates called germ granules is a conserved strategy to posttranscriptionally regulate germline mRNAs. In *Drosophila melanogaster*, mRNAs accumulate in germ granules by forming homotypic clusters, aggregates containing multiple transcripts from the same gene. Nucleated by Oskar (Osk), homotypic clusters are generated through a stochastic seeding and self-recruitment process that requires the 3′ untranslated region (UTR) of germ granule mRNAs. Interestingly, the 3′ UTR belonging to germ granule mRNAs, such as *nanos* (*nos*), have considerable sequence variations among *Drosophila* species and we hypothesized that this diversity influences homotypic clustering. To test our hypothesis, we investigated the homotypic clustering of *nos* and *polar granule component* (*pgc*) in four *Drosophila* species and concluded that clustering is a conserved process used to enrich germ granule mRNAs. However, we discovered germ granule phenotypes that included significant changes in the abundance of transcripts present in species’ homotypic clusters, which also reflected diversity in the number of coalesced primordial germ cells within their embryonic gonads. By integrating biological data with computational modeling, we found that multiple mechanisms underlie naturally occurring germ granule diversity, including changes in *nos*, *pgc*, *osk* levels and/or homotypic clustering efficacy. Furthermore, we demonstrated how the *nos* 3′ UTR from different species influences *nos* clustering, causing granules to have ∼70% less *nos* and increasing the presence of defective primordial germ cells. Our results highlight the impact that evolution has on germ granules, which should provide broader insight into processes that modify compositions and activities of other classes of biomolecular condensate.

## Introduction

**Fig. 1.**
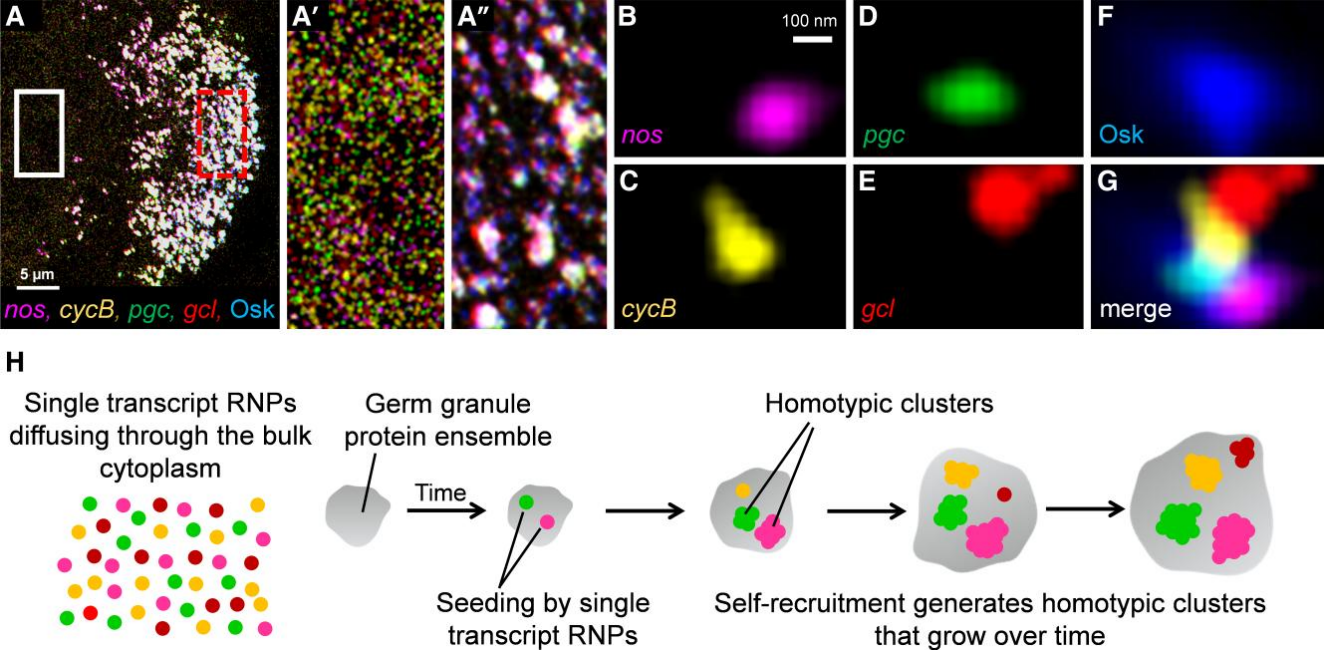
Localization of mRNAs to the germ plasm by forming homotypic clusters within germ granules in *D. mel*. (*A*) Superresolution max project image of germ plasm mRNAs *nos*, *cycB*, *pgc*, and *gcl* that are labeled using smFISH and Osk protein tagged with GFP in a stage 13 oocyte; posterior is to the right. (*A*′) Enlarged region of (*A*) marked by the solid box that depicts the single transcript RNPs that are diffusing through the bulk cytoplasm of the oocyte. (*A′′*) Enlarged region of germ granules (marked by Osk-GFP), in the posterior germ plasm that is marked by the broken box in (*A*). (*B*–*G*) Germ plasm mRNAs that have accumulated within a single germ granule by forming homotypic clusters. (*H*) Overview of homotypic cluster formation in *D. mel* as previously described (Niepielko et al. 2018; Valentino et al. 2022).

Throughout the animal kingdom, germline function and maintenance require the formation of ribonucleoprotein (RNP) granules called germ granules. Specifically, germ granules are biomolecular condensates that function in the posttranscriptional regulation of mRNAs that have critical roles in germline differentiation, proliferation, and function (Seydoux and Braun 2006; Strome and Lehmann 2007; Cinalli, et al. 2008; Ewen-Campen, et al. 2010; Voronina, et al. 2011; Sengupta and Boag 2012; Gao and Arkov 2013; Trcek and Lehmann 2019; Chiappetta, et al. 2022; Thomas, et al. 2023). In *Drosophila*, germ granules, also referred to as polar granules, develop within a highly specialized cytoplasm called the germ plasm that develops at the oocyte's posterior and persists in the early embryo for ∼1–2 h postfertilization (Lécuyer, et al. 2007; Rangan, et al. 2009). The formation of the germ plasm and its germ granules begins when *oskar* mRNA (*osk*) accumulate in founder granules that form at the posterior of the oocyte, where it is translated and recruits additional proteins such as Vasa (Vas) and Tudor (Tud) to form germ granule protein ensembles. Although several proteins are known to comprise the germ granule protein ensemble, Osk is the only protein that is both necessary and sufficient for germ plasm formation (Boswell and Mahowald 1985; Ephrussi and Lehmann 1992; Breitwieser, et al. 1996; Mahowald 2001; Eichler, et al. 2020). Comprising the mRNA portion of germ granules are transcripts such as *germ-cell less* (*gcl*), *cyclinB* (*cycB*), *nanos* (*nos*), and *polar granule component* (*pgc*) (Little, et al. 2015; Trcek, et al. 2015). These genes are maternally transcribed by specialized cells called nurse cells, which then deposit the mRNAs into the oocyte's bulk cytoplasm as RNPs containing single transcripts (fig. 1) (Little, et al. 2015). Simultaneously with the Osk-driven formation of the germ granule protein ensembles, single transcript RNPs that are diffusing through the bulk cytoplasm localize to the germ plasm by incorporating into germ granules through a stochastic seeding and self-recruitment process that results in the formation of homotypic clusters, mRNA aggregates that contain several copies of the same transcript (fig. 1) (Little, et al. 2015; Niepielko, et al. 2018; Valentino, et al. 2022). During homotypic clustering, mRNA abundance within a granule can be dictated by 3′ untranslated region (UTR) sequences, called clustering/localization elements, whereas self-recruitment/self-sorting is independent from such sequences (Eagle, et al. 2018; Trcek, et al. 2020; Valentino, et al. 2022). The formation and growth of germ granules occurs continuously for ∼19 h from oocyte stages (∼9 to 14) to the early embryo, when the degradation of *osk* mRNA has completed (Ephrussi and Lehmann 1992; Niepielko, et al. 2018; Eichler, et al. 2020; Valentino, et al. 2022). Germ granules are ultimately inherited by developing primordial germ cells, known as pole cells in *Drosophila*, and this inheritance supplies the transcriptionally silent cells with maternal mRNAs that direct the production of proteins that are essential to germline function and viability (Cinalli, et al. 2008; Sengupta and Boag 2012). Primordial germ cells, with their inherited germ granule content, initially bud from the posterior pole of the embryo and migrate anteriorly until they ultimately coalesce into the embryonic gonads (Santos and Lehmann 2004).

Recent studies have employed a combination of techniques such as single-molecule fluorescent in situ hybridization (smFISH), superresolution microscopy, quantitative image analysis, and computational modeling to investigate germ granule development by quantifying the mRNA content of germ granules in *Drosophila melanogaster* (*D. mel*) (Little, et al. 2015; Eagle, et al. 2018; Niepielko, et al. 2018; Trcek and Lehmann 2019; Trcek, et al. 2020; Chiappetta, et al. 2022; Valentino, et al. 2022). These interdisciplinary methods have enabled the quantification of the number of transcripts found within a homotypic cluster (referred to as cluster size), the characterization of homotypic cluster size distribution for thousands of clusters found throughout a germ plasm (referred to as germ plasm landscape), the measuring of the frequency at which different types of homotypic clusters populate the same granule (referred to as colocalization), and the description of the relationship between the sizes of different homotypic clusters that are detected within the same granule (Little, et al. 2015; Niepielko, et al. 2018; Valentino, et al. 2022). Such granule features can be collectively obtained and visualized through the Granule Census, an image analysis pipeline that transforms 3D confocal images of smFISH data into a quantified 2D matrix, which can also be produced in silico using a computational model that simulates the nucleation and growth of germ granule protein ensembles and homotypic cluster development (Niepielko, et al. 2018; Valentino, et al. 2022).

The mRNA composition of germ granules relies primarily on three known mechanisms that work in conjunction with each other: 1) the transcript levels of a particular germ granule mRNA; 2) the amount of *osk* present in the germ plasm, which controls the granule's mRNA carrying capacity (cc); and 3) the efficacy by which an mRNA can accumulate within a homotypic cluster, referred as the clustering factor, which has been shown to be influenced by *cis*-acting sequences called “clustering elements” found in the 3′ UTR (Eagle, et al. 2018; Valentino, et al. 2022). Although germ granule formation and mRNA composition has been described and modeled in *D. mel* and several mutants (Little, et al. 2015; Niepielko, et al. 2018; Valentino, et al. 2022), it is unclear how conserved the germ granule formation process is in other *Drosophila* species. Previous studies have demonstrated that the accumulation of *nos* in the germ plasm of *D. mel* can be achieved using the *nos* 3′ UTR from other species despite considerable sequence variations identified in the *nos* 3′ UTR, suggesting conserved mechanisms that participate in germ granule formation (Gavis, et al. 1996). However, questions surrounding the impact that evolution has on germ granule assembly mechanisms, germ granule mRNA compositions, and germ cell development have not been investigated. Our goal was to investigate the evolution and development of *Drosophila* germ granules to gain insight into their diversity and to identify developmental mechanisms that are susceptible to modifications.

With a focus on known germ granule mRNAs *nos* and *pgc*, since clustering factor has been described for both mRNA types (Eagle, et al. 2018; Valentino, et al. 2022), we performed smFISH and carried out quantitative image analysis in *D. mel*, *Drosophila virilis*, *Drosophila pseudoobscura*, and *Drosophila nebulosa* in developmentally early, mid, and late germ plasms. We found that single transcripts diffusing outside of the germ plasm and the formation of homotypic clusters are conserved features during the germ granule assembly process. However, we discovered that the germ granule mRNA composition can be highly diverse among species, including significant variation in cluster sizes of *nos* and/or *pgc* and colocalization rates between different homotypic clusters. Interestingly, varied germ granule mRNA compositions reflected changes in germ cell development, suggesting a link between germ granule diversity and influence on germ cell development. To identify which mechanism(s) generate diversity in germ granule mRNA composition, we integrated biological data with computational modeling and found that changes to multiple and combined mechanisms, including transcript levels of *pgc*, *nos*, *osk* and/or clustering factor, contribute to germ granule diversity. To validate the importance of clustering factor in generating germ granule diversity, we expressed the *nos* 3′ UTRs from *D. pseudoobscura* and *D. nebulosa* in *D. mel* and discovered that different *nos* 3′ UTRs generate phenotypes where homotypic clusters contained ∼70% less *nos* on average and increased the presence of defective primordial germ cells. Together, our findings revealed the conserved and diverse features of *Drosophila* germ granules and demonstrate how germ granule mRNA content is subject to evolutionary changes. Additionally, we highlight multiple genetic mechanisms that are prone to evolutionary modifications that underlie *Drosophila* germ granule diversity. More broadly, our findings may offer insight into the evolution, development, and functional tuning of other types of biomolecular condensates while offering a systems-level view of how mRNA localization mechanisms are evolutionarily modified.

## Results

### Homotypic Cluster Formation Is a Conserved Process in *Drosophila* Species

**Fig. 2.**
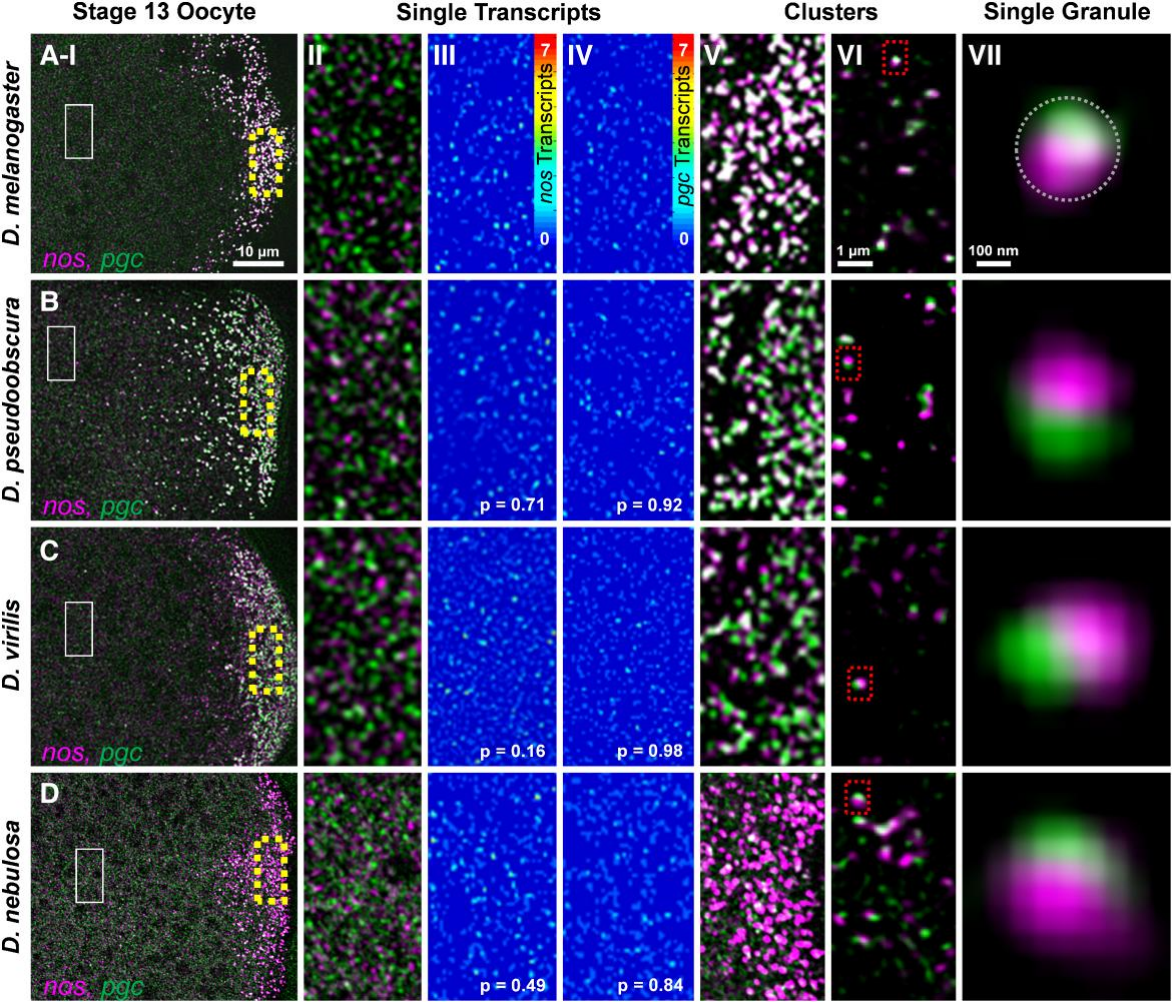
Single transcript RNPs in the bulk cytoplasm and posterior accumulation of *nos* and *pgc*. (Rows *A–D* and column *I*) Confocal images (max projection) of *nos* and *pgc* using smFISH in stage 13 oocytes. (*A*) *D. mel*, (*B*) *D. pse*, (*C*) *D. vir*, and (*D*) *D. neb* (posterior is to the right). (Column *II*) Single transcripts that are not in germ granules and are dispersed throughout the bulk cytoplasm (solid box in column *I*). (Column *III*) Heatmaps of single *nos* molecules in the bulk cytoplasm. (Column *IV*) Heatmaps of single *pgc* molecules in the bulk cytoplasm; *P* values between single-molecule intensities from each species and *D. mel* show no difference (see also Supplementary fig. 1, Supplementary Material online). (Column *V*) Posterior accumulation of *nos* and *pgc* in the germ plasm (broken boxes in column *I*). (Column *VI*) Superresolution images (single slices) of *nos* and *pgc* RNPs in the germ plasm. (Column *VII*) RNPs of *nos* and *pgc* that are spatially determined to be within a single germ granule (marked by the broken circle and broken boxes in column *VI*). Images are a representation of a minimum of three stage 13 germ plasms for each species.

To begin investigating the conservation of germ granule assembly in genus *Drosophila*, we explored 1) whether single RNP molecules of *nos* and *pgc* diffuse in the oocyte bulk cytoplasm and 2) whether each mRNA type generates homotypic clusters in the posterior germ plasm in other *Drosophila* species. We preformed *nos* and *pgc* smFISH experiments, confocal microscopy, and quantitative image analysis on stage 13 oocytes in *D. mel*, *D. pseudoobscura* (*D. pse*), *D. virilis* (*D. vir*), and *D. nebulosa* (*D. neb*). Stage 13 oocytes were chosen based on the presence of both single transcripts in the bulk cytoplasm and large homotypic clusters of *nos* and *pgc* in the posterior germ plasm in *D. mel* (Niepielko, et al. 2018). Similar to *D. mel*, we discovered that RNPs containing single *nos* or *pgc* transcript diffuse through the bulk cytoplasm, outside of the posterior germ plasm in all non-*D. mel* species tested (fig. 2 and supplementary fig. S1*A* and *B*, Supplementary Material online; see Materials and Methods). To identify whether homotypic clustering exists in non-*D. mel* species, we performed quantitative image analysis to calculate how many transcripts reside in the brighter RNPs that were observed in the posterior germ plasm of each species (fig. 2). In *D. mel*, we calculated the average number of transcripts in posterior *nos* RNP*s* was 7.50 ± 0.45 and for *pgc* RNPs was 5.14 ± 0.27 which are both expected values based on previously published work (supplementary fig. S1*C* and *D*, Supplementary Material online) (Niepielko, et al. 2018). For *D. pse*, the average number of transcripts in posterior *nos* RNPs was 4.04 ± 0.34 and for *pgc* RNPs was 5.72 ± 0.98. For *D. vir*, the average number of transcripts in posterior *nos* RNPs was 3.23 ± 0.39 and for *pgc* RNPs was 2.74 ± 0.15. In *D. neb*, the average number of transcripts in the posterior *nos* RNPs was calculated to be 5.32 ± 0.34 and for *pgc* RNPs was 1.49 ± 0.05 (supplementary fig. S1*C* and *D*, Supplementary Material online). In all species, larger *nos* and *pgc* RNPs were also detected (supplementary fig. S1*E* and *F*, Supplementary Material online). Next, we analyzed the frequency that germ plasm *nos* RNPs and *pgc* RNPs reside within the germ granule by calculating their colocalization rates in each species. The colocalization for *D. mel* was 46.38% ± 2.32, which is similar to the expected previously published rate (Niepielko, et al. 2018). In *D. pse*, *D. vir*, and *D. neb*, the colocalization rates between germ plasm *nos* and *pgc* RNPs were 47.06% ± 3.77, 22.00% ± 3.54, and 18.38% ± 4.46 (supplementary fig. S1*G*, Supplementary Material online), respectively, and were all greater than the previously published 10% rate for random colocalization (Niepielko, et al. 2018). In all species tested, the average number of transcripts that reside in germ plasm *nos* RNPs was >3, whereas the colocalization rate between different mRNA types was under 50%. Additionally, larger *nos* and *pgc* RNPs were observed throughout the germ plasm in all species tested (supplementary fig. S1, Supplementary Material online). Together, these data demonstrate that transcripts from *nos* are more likely to colocalize with itself rather than colocalize with *pgc*, supporting a conserved process to localize mRNAs to the germ plasm through the formation of homotypic clusters. *Drosophila* germ granules are estimated to have an average size of ∼300 nm (Mahowald, et al. 1976; Amikura, et al. 2001; Eagle, et al. 2018). Therefore, we visually explored homotypic clustering in other species by employing superresolution confocal microscopy to detect separation of *nos* and *pgc* RNPs within the same germ granule, comparable with *D. mel*, using the average size of a granule (fig. 2). Together, we conclude that similar to *D. mel*, germ granules in other *Drosophila* species contain homotypic clusters and are heterogenous with respect to the types of clusters that they contain and the number of transcripts found in a cluster.

### The mRNA Composition of Germ Granules Is Diverse among Different *Drosophila* Species

**Fig. 3.**
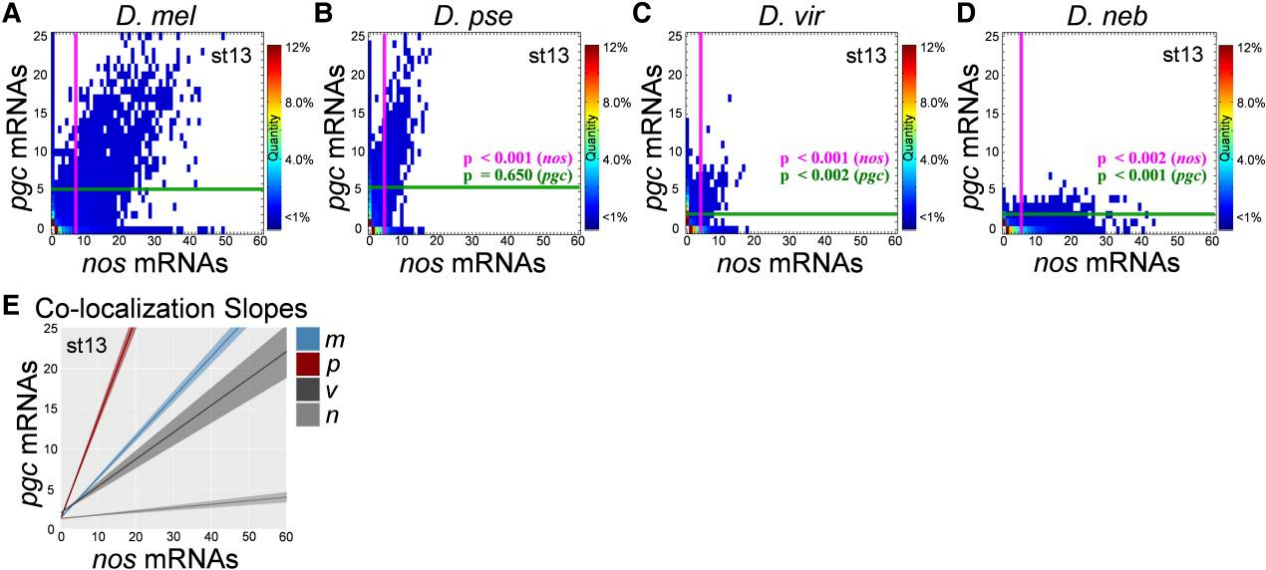
Germ granule censuses produced from various *Drosophila* species. (*A*–*D*) The Granule Censuses produced from analyzing *nos* and *pgc* homotypic clusters in stage 13 germ plasms from *D. mel*, *D. pse*, *D. vir*, and *D. neb*. The vertical line represents the average *nos* cluster size observed in the germ plasm, whereas the horizontal line represents the average *pgc* cluster size in the germ plasm in each census. (*E*) Lines of best fit generated by plotting the cluster sizes of *nos* and *pgc* that reside within the granule (colocalized) for each species. The shaded regions represent the ± SEM for a minimum of three biological replicates. The heatmaps in (*A*–*D*) represent the percent of granules that have a particular *nos* and *pgc* mRNA composition. For all species, *n* > 5,000 homotypic clusters were analyzed, and *P* values were calculated based on comparing each species’ average *nos* or *pgc* clusters size to *D. mel*'s average *nos* or *pgc* cluster size.

We next probed whether germ granule mRNA compositions were diverse among *Drosophila* species by performing a Granule Census analysis on stage 13 oocytes in *D. mel*, *D. pse*, *D. vir*, and *D. neb*. The Granule Census transforms 3D confocal images of germ plasm *nos* and *pgc* smFISH data in a 2D quantified matrix to visualize the mRNA composition of germ granules within the germ plasm landscape (Niepielko, et al. 2018; Valentino, et al. 2022). The Granule Census revealed striking differences between the *nos* and *pgc* compositions of germ granules from *Drosophila* species. Specifically, we found that the average cluster size of *nos* was significantly smaller in all species tested when compared with that in *D. mel P* < 0.002. Additionally, *D. vir* and *D. neb* had significantly smaller clusters of *pgc* when compared with *D. mel* (*P* < 0.002), whereas *D. pse* produced similar sized *pgc* homotypic clusters as *D. mel* (*P* = 0.650) (supplementary fig. S1, Supplementary Material online, and fig. 3). In summary, we find that *vir* had the smallest *nos* cluster sizes, whereas *D. neb* had the weakest germ granule accumulation of *pgc* when compared with *D. mel*. Furthermore, we determined that the colocalization rates between *nos* and *pgc* clusters were significantly lower in *D. vir* and *D. neb* (*P* < 0.001) when compared with those in *D. mel* (supplementary fig. S1*G*, Supplementary Material online). Next, we analyzed the relationship between the sizes of colocalized *nos* and *pgc* clusters by calculating the slope of the best fit line of colocalized cluster sizes in different *Drosophila* species. For *D. mel*, the slope was 0.50 ± 0.02 and consistent with previously published results (Niepielko, et al. 2018). In *D. pse*, the slope increased to 1.24 ± 0.07 (*P* < 0.001) when compared with that in *D. mel*. For *D. vir* and *D. neb*, the slope was reduced to 0.33 ± 0.05 and 0.044 ± 0.0 (*P* = 0.032 and *P* < 0.001), respectively, when compared with that for *D. mel* (fig. 3*E*). Together, these data demonstrate that homotypic clustering, colocalization rates, and the balance between the sizes of clusters that populate the same granule can be diverse or similar among different *Drosophila* species.

**Fig. 4.**
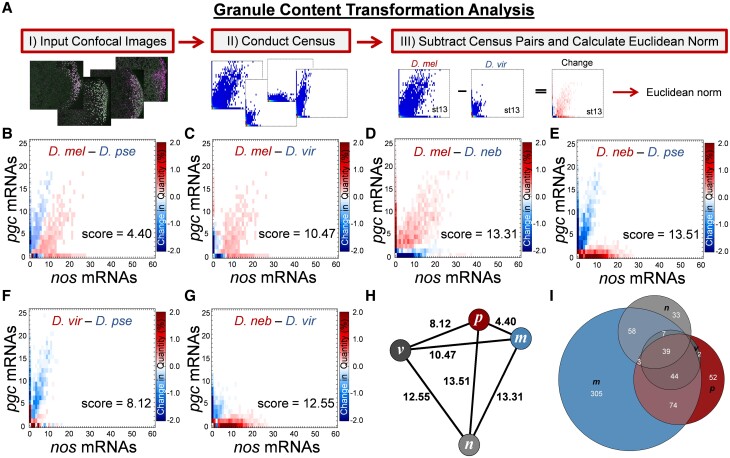
Scoring the overall diversity of *nos* and *pgc* germ granule content. (*A*) Changes in germ granule mRNA content are scored using a pipeline called the GCTAnalysis. The pipeline begins by acquiring 3D confocal images and transforming them into quantified 2D Granule Censuses. Next, a new census that represents a change between two Granule Censuses is generated by subtracting two censuses. To score the overall magnitude of change, the Euclidean norm of the resulting census is calculated. (*B*–*G*) Changes in *nos* and *pgc* germ granule mRNA content between different pairs of *Drosophila* species in stage 13 oocytes with the overall score noted. The heatmap represents a change in quantity for a given *nos* and *pgc* combination found in the Granule Census; the darker the matrix element, the larger the change between the paired species, whereas white represents no change in a particular composition between the different species. (*H*) Undirected graph where nodes are represented by species and edges are represented by the score calculated in (*B*–*G*): *melanogaster* (*m*), *pseudoobscura* (*p*), *virilis* (*v*), and *nebulosa* (*n*). (*I*) Venn diagram that displays the number of *nos* and *pgc* mRNA compositions that are unique or overlapping between different *Drosophila* species.

All germ granule feature measurements can be extrapolated from the Granule Census, including colocalization rates, slopes, cluster sizes, and their distribution (Niepielko, et al. 2018; Valentino, et al. 2022). Rather than focusing on any individual germ granule feature, we aimed to characterize and score the overall change in the *nos* and *pgc* composition of germ granules between *Drosophila* species. To identify overall change between entire Granule Census, we developed a dimension reduction pipeline called the Granule Content Transformation Analysis (GCTAnalysis), which highlights the transformation from one germ plasm to another and provides scores that represent the magnitude of change between two censuses where a larger value refers to a greater transformation (fig. 4*A*). By calculating GCTAnalysis scores for each species pair, we found that the score for *D. mel* to *D. pse* was 4.40, for *D. mel* to *D. vir* was 10.47, *D. mel* to *D. neb* was 13.31, *D. pse* to *D. neb* was 13.51, *D. vir* to *D. pse* was 8.12, and *D. vir* to *D. neb* was 12.55 for stage 13 oocytes (fig. 4*B*–*G*). Next, we generated an undirected graph to visualize the magnitude of change between species’ germ plasms, and in summary, our analysis between *nos* and *pgc* mRNA compositions revealed that the germ plasm between *D. mel* to *D. pse* was most similar, whereas *D. neb* was the most different when compared with the other species (fig. 4*H*). Next, we analyzed the number of *nos* and *pgc* mRNA compositions that are similar or unique among species’ germ plasms. Overall, we found that *D. mel* had the most unique *nos* and *pgc* mRNA compositions found within germ granules at 305, followed by *D. pse* at 52 and *D. neb* at 33. We did not observe any *nos* and *pgc* mRNA compositions that were unique to *D. vir* in stage 13 oocytes (fig. 4*I*). Altogether, our comprehensive analyses show that the mRNA composition of germ granules is subject to evolutionary changes that generate germ plasm diversity among *Drosophila* species.

### Presence of Germ Granule Diversity in Early Stages of Germplasm Formation

**Fig. 5.**
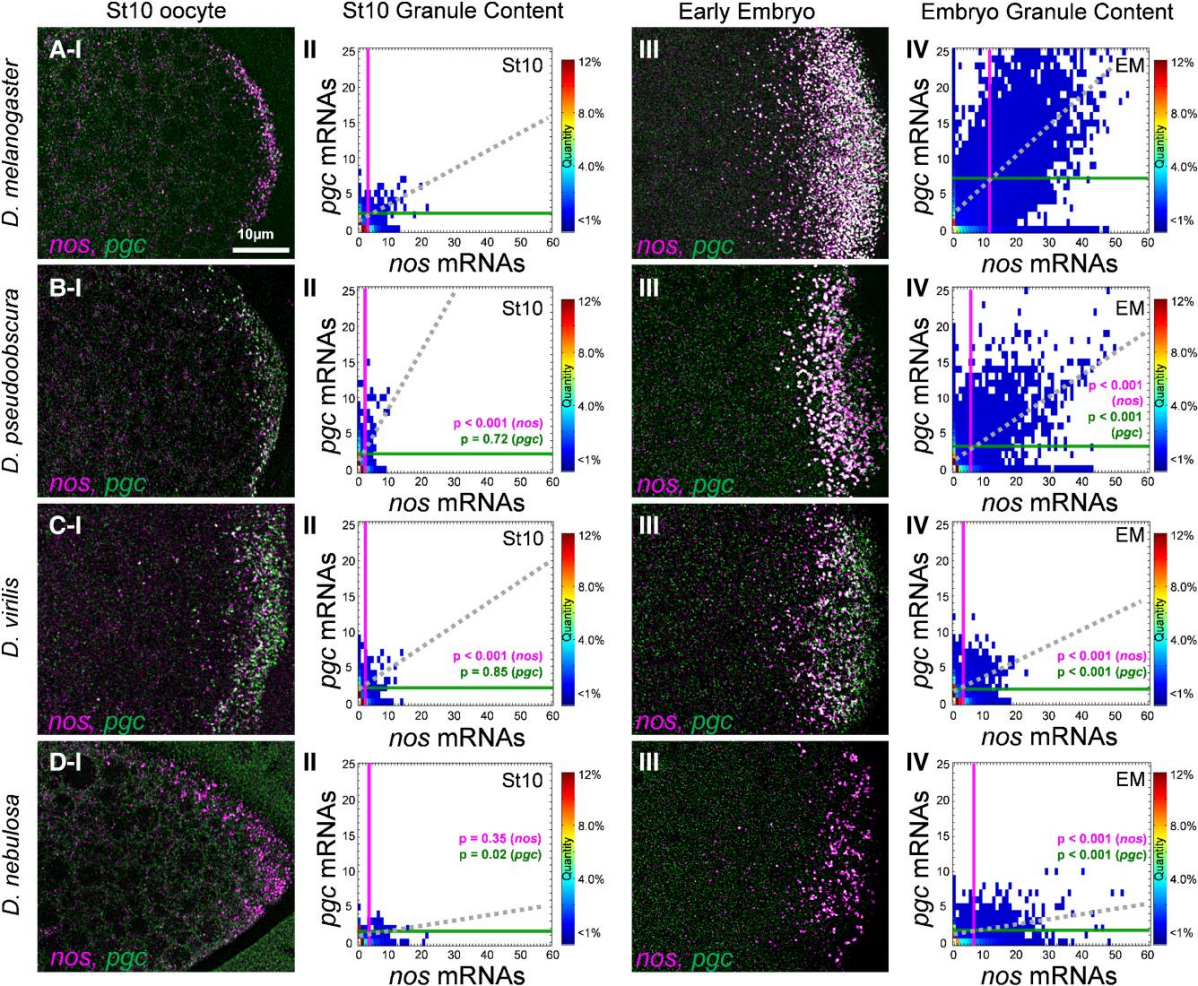
Germ granule mRNA composition at stage 10 and the early embryo in *Drosophila* species. (Rows *A*–*D* and column *I*) Confocal images (max projection) of *nos* and *pgc* using smFISH in stage 10 oocytes. (*A*) *D. mel*, (*B*) *D. pse*, (*C*) *D. vir*, and (*D*) *D. neb* (posterior is to the right). (Column *II*) Granule Census for each species’ germ plasm in stage 10 oocytes. (Column *III*) Confocal images (max projection) of *nos* and *pgc* using smFISH in the early embryo. (Column *IV*) Granule Census for each species’ germ plasm in the early embryo. For all species, images are a representation of a minimum of three stage 10 and early embryo germ plasms. Each stage 10 census includes *n* > 1,300 granules, whereas each early embryo census includes *n* > 10,000 granules. The vertical line represents the average *nos* cluster size observed in the germ plasm, whereas the horizontal line represents the average *pgc* cluster size in the germ plasm in each census. The dotted line represents the line of best fit that is produced by the sizes of colocalized *nos* and *pgc* clusters.

Given the observed germ granule diversity observed among different *Drosophila* species during stage 13 oocytes (figs. 3 and 4), we next examined whether diversity in germ granule mRNA composition can be observed early during germ plasm formation. To explore when germ granule diversity can be detected, we performed smFISH and the Granule Census for *nos* and *pgc* on stage 10 oocytes for *D. mel*, *D. pse*, *D. vir*, and *D. neb*. The average *nos* cluster sizes were 3.14 ± 0.11 for *D. mel*, 2.17 ± 0.09 for *D. pse*, 2.10 ± 0.09 for *D. vir*, and 3.34 ± 0.13 for *D. neb*. On average, *nos* cluster sizes were smaller by only ∼1 transcript in *D. pse* and *D. vir* (*P* < 0.001), whereas *nos* cluster sizes were similar in *D. neb* when compared with those in *D. mel* (*P* = 0.35). For *pgc*, the average cluster size was 2.35 ± 0.17 in *D. mel*, which was not significantly different when compared with that in *D. pse* (2.59 ± 0.08) and *D. vir* (2.17 ± 0.30) (*P* > 0.71). However, diversity in *pgc* homotypic clustering was observed in *D. neb* in that homotypic clusters for *pgc* were not readily detected, with an average cluster size of 1.59 ± 0.05, which was significantly different when compared with that in *D. mel* (*P* = 0.02) (fig. 5). Next, we analyzed the colocalization rates between *nos* and *pgc* in stage 10 oocytes and found that *D. mel* had a colocalization rate of 29.12% ± 3.34, which was similar to previously published data (Niepielko, et al. 2018). This colocalization rate was not significantly different when compared with colocalization rates of *D. pse* (29.00% ± 3.00), *D. vir* (25.00% ± 8.40), and *D. neb* (22.5% ± 2.90) (*P* > 0.55) which were all greater than the 10% random colocalization rate (Niepielko, et al. 2018). Following the calculation of colocalization rates, we analyzed the slope generated by the sizes of colocalized *nos* and *pgc* homotypic clusters for all species in stage 10 oocytes and found that *D. mel* had a slope of 0.25 ± 0.03, which was similar to the slope generated by *D. vir* (0.32 ± 0.07, *P* = 0.63). The slope generated by colocalized stage 10 *nos* and *pgc* clusters in *D. pse* (0.73 ± 0.08) was significantly greater than that in *D. mel* (*P* < 0.001). The slope in *D. neb* stage 10 oocytes was 0.08 ± 0.02, which was significantly less than that in *D. mel* (*P* < 0.03), demonstrating the presence of diversity in the balance between *nos* and *pgc* cluster sizes between *D. mel* and other species (fig. 5). Together, these data show that germ granule diversity arises early in germ plasm formation for some germ granule features.

### Germ Granule Formation Dynamics Vary among *Drosophila* Species

In *D. mel*, *nos* and *pgc* clusters persist in the germ granules of 1–2-h-old embryos (Little, et al. 2015). Thus, we examined whether germ granule diversity is also observed in the early embryo. We first determined that the average germ granule content of *nos* in *D. mel* was 11.13 ± 0.75 and was similar to previously published data (Niepielko, et al. 2018). Next, we found that the average number of *nos* transcripts observed in germ granules for *D. pse* was 5.37 ± 0.14, for *D. vir* was 3.07 ± 0.27, and for *D. neb* was 6.50 ± 0.40, which were all significantly less than the average in *D. mel* (*P* < 0.001; fig. 5). For *pgc*, the average germ granule content in *D. mel* germ granules in the early embryo was 6.56 ± 0.59 and was consistent with previously published data (Niepielko, et al. 2018). The average number of *pgc* transcripts observed in germ granules for *D. pse* was 3.64 ± 0.25, for *D. vir* was 2.23 ± 0.07, and for *D. neb* was 1.60 ± 0.19, which were all significantly less than the average when compared with that for *D. mel* (*P* < 0.001; fig. 5). As for colocalization, we found that in *D. pse*, *nos* and *pgc* had a colocalization of 36.00% ± 3.53; in *D. vir*, it was 28.70% ± 2.00; and in *D. neb*, it was 22.71% ± 5.80. All these values were all significantly lower than the 56.50% ± 1.91 colocalization rate calculated for *D. mel* (*P* < 0.005). Analyzing the slopes created by the sizes of colocalized *nos* and *pgc* clusters, we found that *D. neb* had the smallest slope at 0.04 ± 0.02, followed by *D. vir* at 0.17 ± 0.03, and *D. pse* at 0.36 ± 0.08 (fig. 5). In *D. vir* and *D. neb*, these values were all significantly lower than the 0.49 ± 0.04 slope measured for *D. mel* (*P* < 0.001), demonstrating that the balance between the number of *nos* and *pgc* transcripts can vary between *D. mel* and other species in the early embryo. Together, these data show that in addition to the oocyte germ plasm, diversity in *nos* and *pgc* clustering is also observed in the germ granules of early embryos.

In the early embryo, germ granules in *D. mel* contain more *nos* and *pgc* transcripts than what is observed in stage 13 oocyte germ plasm. Specifically, the average number of *nos* transcripts in *D. mel* germ granules increases from ∼7 to ∼11 from stage 13 oocytes to the early embryo, whereas the average number of *pgc* transcripts increases from ∼6 to ∼7 transcripts, which supports a model of continuous mRNA accumulation for both *nos* and *pgc* (Little, et al. 2015; Niepielko, et al. 2018). Our next goal was to explore whether other species have similar accumulation dynamics and to capture any changes in the behavior of germ granule formation. First, we compared the average cluster sizes of *nos* between stage 13 oocytes and the early embryo in non-*D. mel* species. In *D. pse* and *D. neb*, the observed increase in *nos* cluster size between stages was significant (*P* < 0.044), suggesting that like *D. mel*, these species also continue to accumulate *nos* in the early embryo; however, the increase in *nos* for these species was modest in comparison with the change observed in *D. mel* (figs. 3 and 5). Unlike the other three species, *nos* cluster sizes were static between stage 13 and the early embryo for *D. vir* at ∼3 transcripts. As for *pgc*, cluster sizes were static between stage 13 and the early embryo for *D. neb* at ∼1.5 transcripts and *D. vir* at ∼2 transcripts, suggesting that unlike *D. mel*, *pgc* and/or *nos* accumulation does not continue in the early embryo for these species. Interestingly, a small decrease in *pgc* cluster size was detected from stage 13 and early embryo in *D. pse* (*P* < 0.04), suggesting possible degradation of *pgc* homotypic clusters in the early embryo in *D. pse* (see Discussion).

To measure and visualize overall differences in *nos* and *pgc* accumulation dynamics between each species, we calculated and triangulated GCTAnalysis scores between stages 10, 13, and the early embryo for each species and analyzed the changes in triangle shapes (supplementary fig. S2, Supplementary Material online). In *D. mel*, the scores generated an obtuse scalene triangle, with the obtuse angle of 146° at the stage 13 node. However, in *D. pse* and *D. vir*, the scores generated acute scalene triangles with angles of 81° and 88° at the stage 13 node, respectively. In *D. neb*, the scores also created an obtuse scalene triangle; however, the obtuse angle of 160° was at the early embryo (EM) node, unlike the obtuse angle at the stage 13 node in *D. mel* (supplementary fig. S2, Supplementary Material online). The triangles highlight differences in germ granule formation dynamics such as the lack of change from stage 13 to the embryo in *D. vir* (supplementary fig. S2*C*, Supplementary Material online) and a regression of germ granule composition from stage 13 toward stage 10 in *D. neb* embryos (supplementary fig. S2*D*, Supplementary Material online). By generating species-specific triangles, we were able to visualize how the developmental dynamics of germ granules vary between stages and among species. Together, these data show diversity in the accumulation of *nos* and *pgc* transcripts in the early embryo along with variability in the dynamics of germ granule assembly.

### Computational Modeling Recapitulates Germ Granule Diversity

In *D. mel*, the germ granule accumulation of *nos* and *pgc* can be altered by changing the transcript expression levels of *nos*, *pgc*, and/or *osk*. Furthermore, *cis-*acting elements found in the 3′ UTR also contributes to a transcript-specific “clustering factor,” which represent the efficacy by which a gene's bulk cytoplasm single transcript can incorporate into a homotypic cluster within a germ granule (Eagle, et al. 2018; Niepielko, et al. 2018; Valentino, et al. 2022). We hypothesized that the diversity observed in germ granule mRNA composition among *Drosophila* species (figs. 3–5) is driven by one or a combination of these mechanisms. To test our hypothesis, we first measured the transcript levels of *nos*, *pgc*, and *osk* in *D. pse*, *D. vir*, and *D. neb* and compared each expression with their *D. mel* counterpart (see Materials and Methods). Relative to *D. mel*, we found that *osk* levels, which relates to germ granules’ cc (Valentino, et al. 2022), were not significantly different in *D. neb* (*P* > 0.10). However, *D. pse osk* levels were higher at 2.00 ± 0.30 (*P* = 0.039), whereas *osk* levels in *D. vir* were lower at 0.43 ± 0.10 (*P* = 0.019) (supplementary fig. S3, Supplementary Material online). For *nos* expression levels, *D. pse* and *D. neb* did not show a significant change in expression levels (*P* > 0.69) and only *D. vir* had a lower expression level (0.65 ± 0.08, *P* < 0.04) when compared with *D. mel*. Like *nos* expression, *pgc* in *D. pse* and *D. neb* did not show a significant change in expression levels *P* = 0.99 and only *D. vir* had a lower expression level (0.47 ± 0.08, *P* = 0.007) when compared with *D. mel* (supplementary fig. S3, Supplementary Material online). In *D. vir*, we reason that the reduction in both *nos* and *pgc* cluster sizes, when compared with that in *D. mel*, corresponds to the observed decrease in *nos*, *pgc*, and *osk* levels (figs. 3 and 5 and supplementary fig. S3, Supplementary Material online). However, in *D. pse*, *nos* cluster sizes are reduced despite an increase in *osk* levels and equal level expression of *nos*. Furthermore, in *D. neb*, where the average *nos* clusters are smaller than *D. mel*'s and poor clustering of *pgc* is observed, there are no changes in *nos*, *pgc*, or *osk* levels (figs. 3 and 5 and supplementary fig. S3, Supplementary Material online). These data suggest that changes in expression levels alone cannot account for the evolutionary changes in germ granule mRNA composition for every species, and therefore, we hypothesized that differences in the clustering factor also contribute to germ granule diversity in *D. pse* and *D. neb*.

**Fig. 6.**
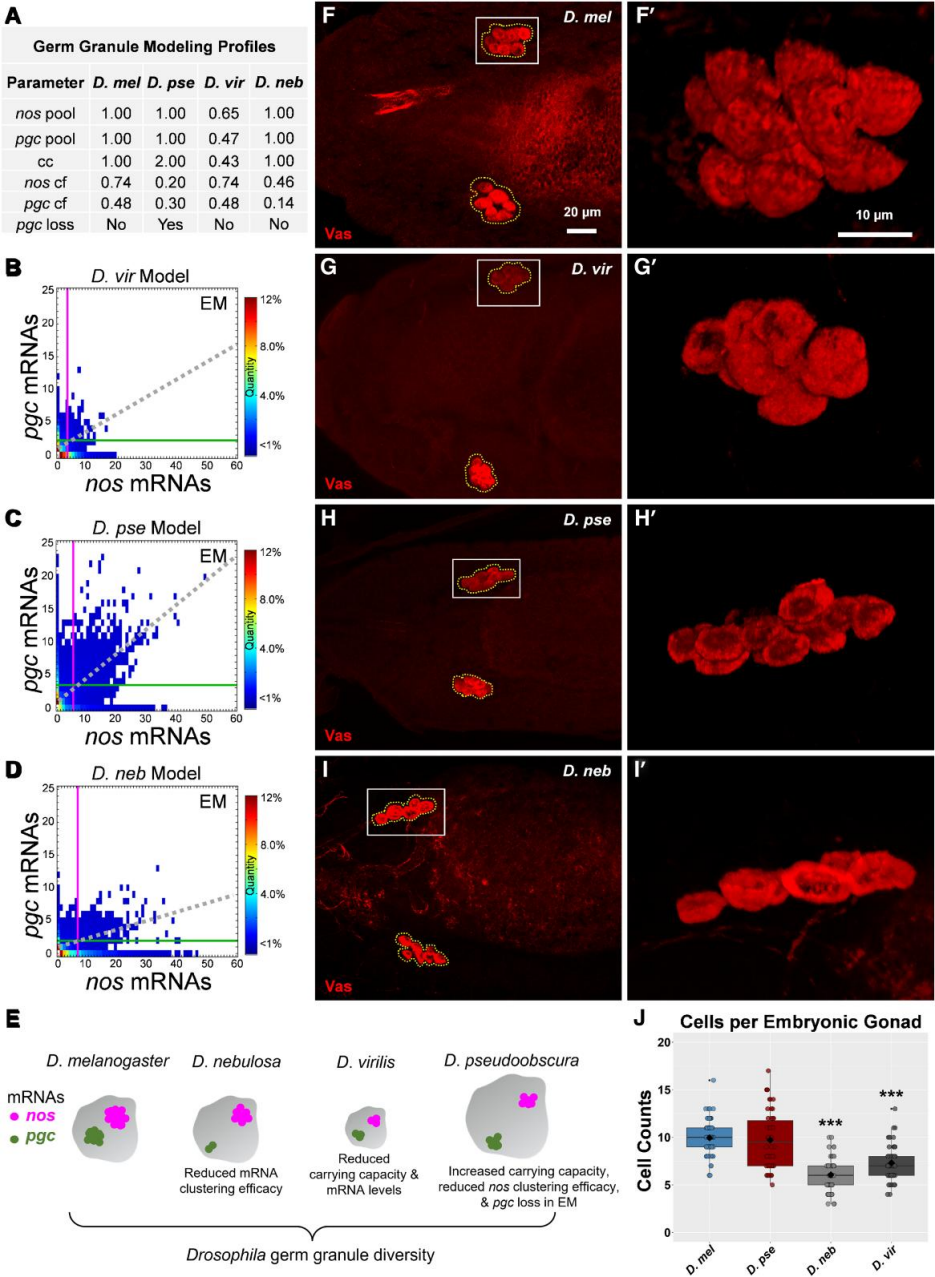
Computational modeling of germ granule diversity and identification of primordial germ cell diversity. (*A*) Germ granule modeling profiles for each species used to generate embryonic germ granules in silico. (*B*–*D*) Germ Granule Censuses produced using computational modeling profiles in (*A*) for (*B*) *D. vir*, (*C*) *D. pse*, and (*D*) *D. neb*. The vertical line represents the average *nos* cluster size observed in the germ plasm, whereas the horizontal line represents the average *pgc* cluster size in the germ plasm in each census. The dotted line represents the line of best fit produced by *nos* and *pgc* cluster sizes that reside within the same granule. (*E*) Cartoon summarizing how germ granule diversity is obtained. (*F*–*I*) Confocal max projection of primordial germ cells, detected with Vas, in *D. mel*, *D. vir*, *D. pse*, and *D. neb*. Primordial germ cells that coalesced in the embryonic gonad are outlined with abroken polygon. (*F′*–*I′*) Enlarged 3*D* rendering of coalesced cells marked by solid boxes in (*F*–*I*). (*J*) The number of primordial germ cells observed per coalesced group of cells for each species: *n* = 72 (36 embryos) for *D. mel*, *n* = 60 (30 embryos) for *D. vir*, *n* = 54 (27 embryos) for *D. pse*, and *n* = 58 (29 embryos) for *D. neb*, *** denotes *P* < 0.001 compared with *D. mel*. The black diamond represents the average.

To measure how changes in clustering factor contribute to the observed germ diversity found in *D. pse* and *D. neb*, we adapted a previously published computational modeling (Valentino, et al. 2022) and calculated the clustering factor of *nos* and *pgc* in non-*D. mel* species. Specifically, we adjusted the model's transcript pool parameters to the relative expression levels of *nos* and *pgc* and set the germ granule's cc parameter to the relative *osk* level from each species. To predict clustering factors for *nos* and *pgc* in *D. pse* and *D. neb*, we first added a loss parameter to *pgc* to the adapted *D. pse* computational model which represented the observed decrease in *pgc* cluster size observed from stage 13 to early embryo (see Materials and Methods). Next, we fitted the average cluster size to a clustering factor standard curve (supplementary fig. S4*A* and *C*, Supplementary Material online, and see Materials and Methods). We found that in *D. pse*, the *nos* clustering factor was 0.20, whereas the *pgc* clustering factor was 0.30. In *D. neb*, we calculated the *nos* and *pgc* clustering factors to be 0.46 and 0.14, respectively (supplementary fig. S4*A* and *C*, Supplementary Material online). In each case, the clustering factors are smaller than the previously determined values of 0.74 for *nos* and 0.48 for *pgc* in *D. mel* (Valentino, et al. 2022). Thus, in cases where clustering factor is reduced but expression levels are unchanged, the unlocalized pool of single transcript in the bulk cytoplasm will be less depleted due to reduced homotypic clustering. Next, we generated modeling profiles for each species where the parameters are represented by the observed expression levels and the calculated clustering factors (fig. 6*A*). Using the modeling profiles, we adjusted the model to recreate each species’ biological conditions in silico and simulated germ granule formation that recapitulated the Granule Census in the early embryo (fig. 6). Using the model's accuracy score (Valentino, et al. 2022), we determined that *D. vir*'s model was 91% accurate, *D. pse* model's was 81% accurate, and *D. neb*'s model was 80% accurate, when compared with their biological counterparts. Using the computational model, we were able to animate Granule Censuses over developmental time and captured how species’ germ granule development varies significantly throughout germ plasm development (supplementary movies 1–3, Supplementary Material online). Together, our biological and modeling data provide system-level evidence that multiple and combined mechanisms contribute to the evolution and development of *nos* and *pgc* compositions in *Drosophila* germ granules.

### Diversity in the Number of Primordial Germ Cells among *Drosophila* Species

In *Drosophila*, primordial germ cells develop from the germ plasm and initially bud from the posterior embryo and migrate anteriorly, eventually aligning and coalescing into the embryonic gonads (Santos and Lehmann 2004). We explored whether diversity in germ plasm reflects changes in germ cell development by quantifying primordial germ cells that coalesce into embryonic gonads using Vasa labeling, immunofluorescence (IF), and confocal microscopy (see Materials and Methods). In *D. mel*, we found an average of ∼10 primordial germ cell in each embryonic gonad (fig. 6), which is consistent with the reported value (Dansereau and Lasko 2008). In *D. pse*, we also observed an average of ∼10 coalesced cells (*P* = 0.97). However, in *D. vir* and *D. neb*, the number of coalesced cells is smaller when compared with that in *D. mel* (*P* < 0.001), with an average of ∼7 cells found in *D. vir* and an average of ∼6 cells in *D. neb* (fig. 6). Interestingly, these species’ Granule Censuses are the most divergent from *D. mel* (fig. 4), suggesting a link between naturally occurring germ granule variations and changes to species’ germline development.

### Sequence Diversity in the *nos* 3′ UTR Yields Differences in Homotypic Clustering

**Fig. 7.**
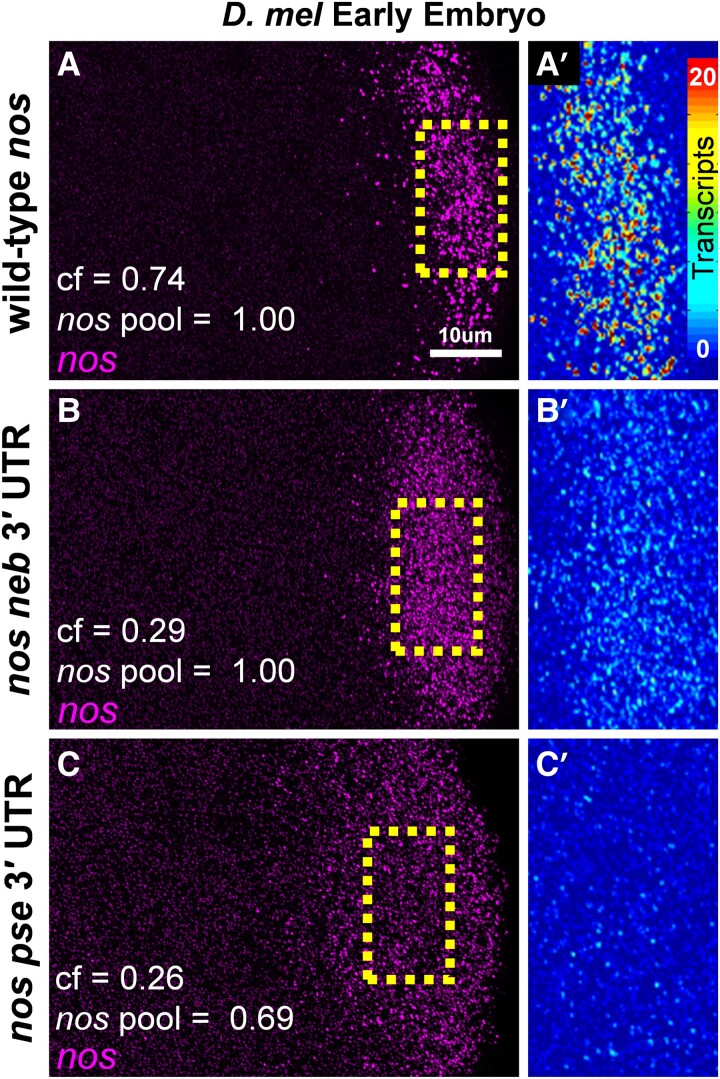
Evolutionary changes in the *nos* 3′ UTR influence the efficacy of *nos* homotypic clustering. (*A*–*C*) Confocal images (max projection) of *nos* using smFISH in the early embryo germ plasm. (*A*) Wild-type *D. mel*, (*B*) *D. mel* with the *nos* 3′ UTR from *D. neb* (*nos neb* 3′ UTR), and (*C*) *D. mel* with the *nos* 3′ UTR from *D. pse* (*nos pse* 3′ UTR). Images are a representation of a minimum of four germ plasms. Cf values and *nos* expression levels (*nos* pool) for each genotype are reported in each panel. (*A′*–*C′*) Enlarged regions of the broken boxes in (*A*–*C*) shown as heatmaps set to the same scale.

Although a combination of changes to the expression levels of *nos*, *pgc*, and *osk* can account for the observed mRNA compositions of germ granules in *D. vir* (fig. 6*A* and *B*), our computational modeling predicts that a reduction in clustering factor contributes to the observed mRNA compositions of germ granules in *D. pse* and *D. neb* (fig. 6). In *D. mel*, the clustering factor for *nos* is regulated, in part, by sequences found in the 3′ UTR (Eagle, et al. 2018; Valentino, et al. 2022). Interestingly, aligning the *nos* 3′ UTRs between *D. mel*, *D. pse*, and *D. neb* revealed considerable sequence variation, resulting in evolutionary distances of 1.06 between *D. neb* and *D. mel*, 0.67 between *D. pse* and *D. mel*, and 0.88 between *D. neb* and *D. pse* (see Materials and Methods). Thus, we hypothesized that differences within the *nos* 3′ UTR can alter *nos* homotypic clustering by reducing *nos*'s clustering factor (fig. 6*A*). To test our hypothesis, we generated *D. mel* flies to have *nos* expressed with the 3′ UTR of either *D. pse* (*D. mel ^pse^*^*nos* 3′ UTR^) or *D. neb (D. mel ^neb^*^*nos* 3′ UTR^) and investigated whether each 3′ UTR could 1) rescue *nos* localization to the germ plasm by forming homotypic clusters and 2) reduce *nos* cluster sizes by lowering *nos*'s clustering factor. To answer these questions, we preformed smFISH, confocal microscopy, and quantitative image analysis on *nos* in the early embryos collected from *D. mel ^pse^*^*nos* 3′ UTR^ and *D. mel ^neb^*^*nos* 3′ UTR^ flies. We found that the *nos* 3′ UTR from *D. pse* and *D. neb* rescues the localization of *nos* to the posterior germ plasm by forming homotypic clusters (fig. 7). Next, we calculated the average cluster sizes of *nos* in both genotypes and compared them with wild-type *nos*. In *D. mel ^pse^*^*nos* 3′ UTR^ and *D. mel ^neb^*^*nos* 3′ UTR^, the average cluster sizes were 2.66 ± 0.23 and 3.45 ± 0.83, respectively. These cluster sizes were significantly different from wild-type *D. mel* which had an average *nos* cluster size of 11.16 ± 0.76 (*P* < 0.0001) (fig. 7). Next, we measured the relative expression levels of chimeric *nos* transcripts and found that the *nos* in *D. mel ^neb^*^*nos* 3′ UTR^ had equal expression to that of wild-type *D. mel* (*P* = 0.99). In *D. mel ^pse^*^*nos* 3′ UTR^, we found that the *nos* transcripts levels were reduced to 0.69 ± 0.05 when compared with those in wild-type *D. mel* (*P* = 0.033) (supplementary fig. S4*D*, Supplementary Material online). Although we observe only 69% of *nos* transcripts in *D. mel ^pse^*^*nos* 3′ UTR^ flies, this reduction cannot account for all of the 76.20% decrease in average cluster size since 63% expression only has a 46.45% decrease (Niepielko, et al. 2018; Valentino, et al. 2022). Thus, we hypothesized that the *nos* 3′ UTRs from different *Drosophila* species reduced cluster sizes by reducing *nos*'s clustering factor. To test our hypothesis, we calculated the clustering factor of the *D. neb nos* 3′ UTR and *D. pse nos* 3′ UTR in *D. mel* by fitting the average cluster sizes to a standard curve generated by the germ granule computational model (supplementary fig. S4*E* and *F*, Supplementary Material online). We determined that the clustering factor for the *D. neb nos* 3′ UTR was 0.29, whereas the clustering factor for *D. pse nos* 3′ UTR was 0.26 when expressed in *D. mel*, resulting in an overall reduction in *nos* localization (fig. 7). In both cases, the clustering factor is reduced when compared with the wild-type 0.74 value (Valentino, et al. 2022). Next, we measured the impact that clustering factor has on the overall localization of *nos* to the germ plasm by comparing the distribution of *nos* cluster sizes between wild-type *D. mel* and *D. mel ^neb^*^*nos* 3′ UTR^ and determining that the two distributions only have 38% overlap (supplementary fig. S4*G*, Supplementary Material online). Our combined modeling and experimental data demonstrate that sequence variation within the *nos* 3′ UTR can diversify *nos* homotypic clustering, indicating that the evolution of noncoding sequences influences germ granule development.

### Different *nos* 3′ UTRs Increase the Presence of Defective Primordial Germ Cells

**Fig. 8.**
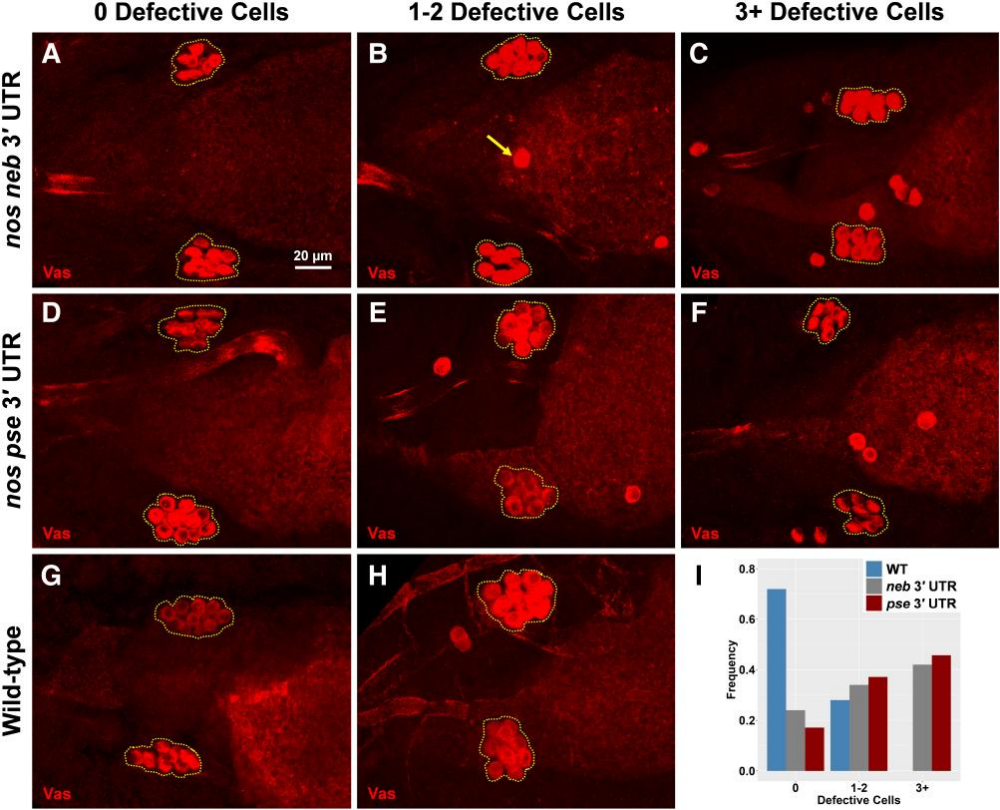
Different *nos* 3′ UTRs increase the presence of defective primordial germ cells. (*A*–*H*) Embryos from *D. mel* with the *nos* 3′ UTR from *D. neb* (*nos neb* 3′ UTR), embryos from *D. mel* with the *nos* 3′ UTR from *D. pse* (*nos pse* 3′ UTR), and embryos from wild-type *D. mel* where 0 defective cells are observed, one to two defective cells are observed, and three or more defective cells are observed. In all max projection images, primordial germ cells are marked with Vas and coalesced primordial germ cells are outlined with a dotted polygon. The arrow in (*B*) highlights an example of defective primordial germ cells, determined by being located outside the broken polygon. (*I*) Fraction of embryos observed for each phenotype: *n* = 38 for *neb* 3′ UTR, *n* = 35 for *pse* 3′ UTR, and *n* = 36 for wild-type *D. mel*.

Primordial germ cells that lack *nos* activity do not properly migrate into the gonads and, therefore, do not become functional germ cells (Kobayashi, et al. 1996). Given that the *nos* 3′ UTRs from different species significantly reduced *nos*'s ability to populate germ granules, we explored whether this change was sufficient to alter the functional role that *nos* has in ensuring robust primordial germ cell migration. By visualizing primordial germ cells after they have coalesced into the gonads, we identified and quantified the number of cells that did not migrate properly, referred to as defective cells. In the wild type, we found that 72% of embryos had no defective cells, whereas the other 28% had only one or two. However, in *D. mel ^neb nos^*^3′ UTR^ and *mel ^pse nos^*^3′ UTR^, 42% and 46% of their embryos had phenotypes of three or more defective cells (up to nine), respectively (fig. 8). Although we observe a smaller number of primordial germ cells in certain species (fig. 6), we found that the average number of coalesced cells was ∼10 in *D. mel ^neb nos^*^3′ UTR^ and *mel ^pse nos^*^3′ UTR^, which was the same as the wild type (*P* > 0.28) (supplementary fig. S4*H*, Supplementary Material online), suggesting that the *nos* 3′ UTR is not sufficient to recapitulate the coalesced phenotype observed in *D. neb* and that additional mechanisms that contribute to changing germ cell development are present (see Discussion). Nonetheless, our findings highlight how evolutionary differences in the *nos* 3′ UTR sequence influence clustering factor, resulting in germ granules with altered *nos* compositions that generate an increased presence of defective primordial germ cells.

## Discussion

Although it is known that *nos* localizes to the posterior germ plasm in other *Drosophila* species such as *D. vir* (Curtis et al. 1995; Gavis et al. 1996), the analysis of *Drosophila* germ granule composition and assembly has been limited to a single species, *D. mel* (Little, et al. 2015; Trcek, et al. 2015; Eagle, et al. 2018; Niepielko, et al. 2018; Trcek, et al. 2020; Valentino, et al. 2022). Despite those studies providing much needed insight into the components, mRNA composition, and mechanisms that underlie germ granule formation, questions such as to what extent are those features conserved and how has the developmental process changed throughout evolution can only be answered by surveying additional species. Recent analysis of the germ plasm from zebrafish showed that similar to *D. mel*, homotypic clusters of *nanos3* and other mRNAs are present and their formation also requires a master organizing protein (Bontems, et al. 2009; Eno, et al. 2019). The appearance of homotypic clustering across phyla introduces intriguing questions regarding the impact that evolution has on germ granule content and the mechanisms that influence their assembly and compositions. Using genus *Drosophila*, we address these evolutionary questions by conducting a comprehensive cross-species analysis that explores conserved and diverse features of germ granules and germ cell development. In doing so, we revealed how evolution impacts germ granule features while identifying genetic mechanisms in the assembly process that are susceptible to modifications.

We found that in *D. vir*, the differences in germ granule composition, when compared with those in *D. mel*, are driven by a combination of reduced *nos*, *pgc*, and *osk* levels. In the computational model, we represent such differences by adjusting parameters that mimic the transcript pools of all three transcripts by reducing their transcription rates (fig. 6*A*). Thus, we reason that differences in homotypic clustering can be attributed to variations at the transcriptional level. Alternatively, mRNA localization in the oocyte relies on mechanisms that protect mRNA from maternal degradation (Lasko 2012; Kara, et al. 2023). Therefore, differences in maternal degradation may also contribute to the disparities in transcript levels among species. In *D. pse* and *D. neb*, the *nos* 3′ UTR is reducing *nos*'s ability to accumulate within germ granules, as measured by the clustering factor value (figs. 6 and 7). In *D. mel*, the sorting of *nos* and *pgc* into different homotypic clusters is not regulated by the 3′ UTR (Trcek, et al. 2020). Rather, the 3′ UTR regulates the size of a cluster, whereas self-sorting is achieved by currently unknown mechanisms (Valentino, et al. 2022). In this study, our data are consistent with this model by further demonstrating how different *nos* 3′ UTRs influence *nos*'s clustering factor. However, a self-sorting role for the 3′ UTR in other *Drosophila* species was not investigated and should be explored. Although our modeling results successfully predicted a decrease in clustering value in *D. pse* and *D. neb*, the predicted values were not equal to the values determined when 3′ UTRs were expressed in *D. mel* (figs. 6 and 7). In *D. mel*, the germ granule protein ensemble contains proteins such as Osk, Vas, and Tud (Boswell and Mahowald 1985; Ephrussi and Lehmann 1992; Breitwieser, et al. 1996; Mahowald 2001). Furthermore, Osk interacts physically with germline mRNAs such as *nos*, *pgc*, and *gcl* and the *D. mel nos* 3′ UTR has been shown to bind to the *C*-terminal lipase-fold domain of Osk (Jeske, et al. 2015; Yang, et al. 2015). Thus, it is possible that germ granule proteins may also contribute to the clustering factor value and that coevolution between 3′ UTRs and germ granule proteins has occurred, generating discrepancies between predicted and experimental clustering values that were calculated when 3′ UTRs were expressed in different species. Indeed, germ granule protein sequences have variations with Osk averaging 0.57 amino acid substitutions per site, whereas Vas and Tud average 0.43 and 0.35 substitutions per site, respectively (supplementary fig. S5*A*, Supplementary Material online). Providing additional support for the coevolution between 3′ UTRs and germ granule proteins is the finding that Osk from *D. vir* cannot concentrate enough germ cell mRNA determinants at the posterior to rescue primordial germ cell formation in *D. mel* (Webster, et al. 1994). In other systems, only a single mutation within the RNA-binding protein fused in sarcoma (FUS) was sufficient to induce structural changes to its condensates (Rhine, et al. 2020), further providing evidence for a potential role for changes in RNA-binding proteins contributing to altered condensate structures. In this study, we focused on the mRNA portion of the germ granule and whether evolutionary changes within germ granule proteins are also contributing to the differences highlighted in this study cannot be ruled out. Understanding how the protein portion of germ granules are changing and whether there is coevolution with specific 3′ UTRs should be addressed in follow-up studies.

We report variability in the number of primordial germ cells that coalesce in the embryonic gonad in species with highly divergent germ plasms, suggesting a link between the two (figs. 4 and 6). However, our study cannot conclude whether changes in *nos* and/or *pgc* compositions alone account for germ cell variability. Specifically, the mRNA from an estimated 61 genes localize to the germ plasm in *D. mel* (Rangan, et al. 2009), including four that have been evaluated for homotypic clustering, *nos*, *pgc*, *gcl*, and *cycB* (fig. 1) (Chiappetta, et al. 2022). Here, we analyzed *nos* and *pgc* since clustering factors have been calculated for both mRNA types in *D. mel* (Eagle, et al. 2018; Valentino, et al. 2022). Although we demonstrate how changes in the *nos* 3′ UTR influence *nos*'s germ granule accumulation, the 3′ UTRs from *pgc*, *gcl*, and *cycB* also have considerable sequence variability, with base substitutions per site averaging 1.09 for *pgc*, 0.95 for *gcl*, and 1.03 for *cycB* among the four species (supplementary fig. S5*B*, Supplementary Material online). Thus, future cross-species studies should focus on gaining a more general sense of how germ granule mRNA content evolves by exploring whether evolutionary changes in other 3′ UTRs also influence germ granule content and germ cell development. In *D. mel*, the mRNA of the master organizer of germ granules, *osk*, also forms homotypic clusters (Little, et al. 2015). However, this occurs in different germ plasm condensates called founder granules which are mutually exclusive with germ granules (Eichler, et al. 2020). Here, our initial interspecies germ plasm study only focuses on the homotypic clustering of germ granule mRNAs. Whether diversification occurs in founder granules that connects to changes in germ granules has not been tested and should be explored in future germ plasm studies. In *D. mel*, a subgranule organization for homotypic clusters has been described in that larger clusters tend to be located centrally within the granule (Trcek, et al. 2015; Chiappetta, et al. 2022). Although no connection has been identified between the geometric order of mRNAs within the germ granule and germline development (Trcek, et al. 2015), our results where a species’ *nos* and *pgc* clusters tend to be of similar size (supplementary fig. S1*C* and *D*, Supplementary Material online, and fig. 5) raise interesting questions regarding the conservation of germ granule subgranule organization, whether organization of subdivided mRNA clusters can exist in species where there is little to no cluster size differential, and if there is influence on germ cell formation. Colocalization rates can also vary among species (supplementary fig. S1, Supplementary Material online). Whether such variation arises due to changes in germ granule subgranule organization and/or changes in the spatial arrangement of different mRNA clusters within the germ plasm cannot be ruled out. Further interspecies analysis into the conservation of subgranule and germ plasm organization may provide additional insight into germ cell development. Together, our study offers a potential link between germ granule diversity and changes to germ cell development, opening the door for exciting new areas of research that investigate how evolutionary changes in germ plasm influence germ cell development.

Besides germline development, *nos* has an essential role in abdomen formation and the lack of posterior localization of *nos* causes embryonic lethality (Lehmann 1988; Wang and Lehmann 1991; Gavis and Lehmann 1992). Here, despite the significant decrease in the germ granule accumulation of *nos* in *D. mel ^neb nos^*^3′ UTR^ and *D. mel ^pse nos^*^3′ UTR^ (fig. 7), these embryos can develop (fig. 8), demonstrating that there is enough *nos* localization and translation to rescue abdomen development. However, increased defective primordial germ cells were observed in both *D. mel ^neb nos^*^3′ UTR^ and *D. mel ^pse nos^*^3′ UTR^ (fig. 8). Interestingly, it has been shown that primordial germ cells that lack *nos* are unable to attenuate the cell cycle and instead continue dividing, leading to an increase of defective primordial germ cells (Deshpande, et al. 1999). Moreover, primordial germ cells that have developed from germ plasm without *nos* localization but retained some Nos activity through mutations in translational regulation tend to not enter mitotic quiescence, resulting in embryos with defective primordial germ cells (Gavis, et al. 2008). We speculate that in *D. mel ^neb nos^*^3′ UTR^ and *D. mel ^pse nos^*^3′ UTR^ embryos, the defective primordial germ cell phenotype may be caused by mitotic defects initiated by insufficient Nos activity from the inheritance of germ granules with reduced *nos* accumulation. Supporting this idea is the recent finding that premature degradation of germ granule mRNAs, such as *cycB*, generates similar defective primordial germ cells (Hakes and Gavis 2023). In previous work, it has been demonstrated, through translational regulation mutants, that *nos* localization can be made dispensable for abdominal patterning but not for germ cell development (Gavis, et al. 2008). Consistent with those findings, our data suggest that this may also be accomplished using a different mechanism, by tuning the *nos* composition of germ granules via 3′ UTR clustering factor to localize enough *nos* for abdomen formation but not enough to avoid increased defective primordial germ cells (fig. 8). Thus, our data present a developmental role for the clustering factor parameter and provide quantitative insight into the localization threshold that *nos* requires to carry out various developmental functions in *D. mel*. Further investigation into how clustering factor may modulate activity thresholds to achieve different biological activities should be the focus of future studies and explored in other biomolecular condensates.

The overall dynamics of homotypic cluster growth vary among *Drosophila* species (supplementary fig. S2 andsupplementary movies 1–3, Supplementary Material online). In *D. vir*, we observed no significant growth in *nos* and/or *pgc* homotypic clusters, generating a mostly static germ plasm landscape from stage 13 to early embryo despite the detection of single transcripts outside of the germ plasm (fig. 5*C* and supplementary fig. S2*C* and supplementary movie 1, Supplementary Material online). These data suggest that *D. vir* germ granules may reach their cc earlier than the other species and support a model where *D. vir* germ granules have a smaller cc when compared with other species (fig. 6). In *D. neb*, *pgc* homotypic clusters were not readily observed in stage 10 oocytes and only developed smaller clusters that do not grow from stage 13 to the early embryo (fig. 5*D* and supplementary fig. S2*D* and supplementary movie 3, Supplementary Material online). Additionally, *pgc* clusters decrease in average cluster size in *D. pse* from stage 13 to early embryo (figs. 3*B* and 5*B* andsupplementary movie 2, Supplementary Material online). In *D. mel*, germ granules are known to have functional plasticity in that they can target certain mRNAs for degradation while preserving protection of others (Hakes and Gavis 2023). Thus, one possible scenario that could explain a decrease in *pgc* cluster sizes from stage 13 to the early embryo in *D. pse* is that it may be targeted early for translation and degradation during the maternal-to-zygotic transition. In *D. mel*, *pgc* functions in establishing transcriptional quiescence in primordial germ cells to help maintain their a germline fate (Deshpande, et al. 2004; Martinho, et al. 2004; Cinalli, et al. 2008; Hanyu-Nakamura, et al. 2008). However, complete loss of *pgc* does not fully prevent the specification of primordial germ cells in *D. mel*, suggesting that *pgc* has an important, but not essential, role in maintaining cell's germline fate due to potentially overlapping or redundant mechanisms (Deshpande, et al. 2004; Deshpande, et al. 2012). Smaller *pgc* cluster sizes that were observed in non-*D. mel* species could signify the presence of additional mechanisms to maintain transcriptional quiescence in primordial germ cells, therefore eliminating a reliance on the formation of larger *pgc* clusters in those species. Interestingly, supplying additional Nos protein to primordial germ cells has been shown to rescue primordial germ cell viability in *pgc* mutants (Deshpande, et al. 2012), suggesting that translational regulation can serve as a mechanism to compensate for reduced abundance of *nos* and/or *pgc* within non*-D. mel* species to ensure primordial germ cell viability. Nonetheless, our study highlights differences in mRNA localization in the germ plasms across multiple *Drosophila* species, and whether there are redundancies and/or translational mechanisms that compensate for different germ granule compositions to ensure germline viability should be investigated in future germ plasm studies.

In genus *Drosophila*, *D. mel*, *D. pse*, and *D. neb* are categorized in subgenera *Sophophora*, with *D. mel* located in the *melanogaster* group, *D. neb* in the *willistoni* group, and *D. pse* in the *obscura* group. *D. vir* is organized within the *virilis* group located in subgenera *Drosophila* (O'Grady 2006; Niepielko, et al. 2011). Our results show that the germ plasm landscape in the late oocyte is most divergent between *D. neb* and the two species found in subgenus *Sophophora* (fig. 4*H*). Thus, the *nos* and *pgc* composition found within germ granules does not fall within the expected phylogenetic relationship. As other mRNAs and species are included from additional studies, it is possible that a more accurate phylogenetic relationship will emerge. Alternatively, the rapid evolution of 3′ UTRs has been implicated as an important genomic meta-regulator that has contributed to the adaptation, diversification, and speciation of cichlid fishes (Xiong, et al. 2018). Thus, it may be that the noncoding genetic components that participate in germ granule assembly, such as 3′ UTRs, are diverging relatively quickly, producing an unexpected phylogenetic picture when comparing germ plasms due to functional impacts on germ granule compositions. Regardless, we provide evidence that the evolutionary divergence of 3′ UTRs can influence germ granule mRNA compositions and function. These discoveries spark curiosity about the wider influence that evolution has on biomolecular condensate structures, the fine-tuning of condensate function, and whether condensate diversity offers natural selection advantages. Further investigation is needed to explore such possibilities. The goal of this initial study was to shed light on evolutionary changes in germline development within the genus *Drosophila* and to identify mechanisms that contribute to the natural diversification of germ granules. Additional classes of biomolecular condensates control various cell processes, including stress response, neurological function, cell signaling, and gene regulation (Brangwynne, et al. 2009; Anderson, et al. 2015; Wheeler and Hyman 2018; Ivanov, et al. 2019; Hayashi, et al. 2021; Jaqaman and Ditlev 2021). Thus, future studies should explore whether the mechanisms described here can be applied to other systems to gain additional knowledge regarding the evolution and development of biomolecule condensates.

## Materials and Methods

### smFISH, Immunofluorescence, and Microscopy

Custom species-specific smFISH probes were designed using Biosearch Technologies Stellaris Probe Designer tool and purchased from Biosearch Technologies where all *pgc* probe sets were labeled with Quasar 570 dye, and all *nos* probe sets were labeled with ATTO 647N dye. Probe designs for *pgc* were based on the full transcript length for *D. mel* (FlyBase, FBgn0016053), UniProt entries A0A6I8V605 for *D. pse*, and B4LMX3 for *D. vir*. Probe designs for *nos* were based on the coding sequence for all species, FBgn0002962 for *D. mel* (FlyBase), UniProt entry A0A6I8V5B3 for *D. pse*, and Q24710 for *D. vir*. For *D. neb*, the *nos* and *pgc* sequences were identified using the *D. neb* annotated long-read sequenced genome (Sottolano, et al. 2022). For *D. mel*, *cycB* and *gcl* probes targeting their coding sequence were generated based on FlyBase sequences FBgn0000405, labeled with CAL Fluor Red 590 dye, and FBgn0005695, labeled with CAL Fluor Red 610 Dye, respectively. smFISH experiments were performed as previously described (Little, et al. 2015; Abbaszadeh and Gavis 2016; Niepielko, et al. 2018), whereas IF was carried out as previously described (Niepielko, et al. 2012; Niepielko, et al. 2014) using polyclonal anti-Vasa (Boster Bio, cat #DZ41154) and secondary Alexa Fluor 568 (anti-rabbit, ThermoFisher, cat #A10042) to label primordial germ cells. In summary, females were fed yeast for 24 h and ovaries were dissected in cold phosphate buffered saline (PBS). For embryo collections, flies were placed in fly cages and allowed to lay eggs for 1 h on apple juice agar plates with yeast paste for smFISH experiments, whereas flies were allowed to lay eggs for 24 h for primordial germ cell staining with anti-Vasa, a primordial germ cell marker (Lasko and Ashburner 1990). Eggs were collected, 0–1-h-old embryos for smFISH and 24 h for IF, then dechorionated and devitellinized as previously described (Abbaszadeh and Gavis 2016). Tissue fixation was carried out for 30 min in 4% paraformaldehyde in PBS. All samples were mounted in ProLong Glass (Life Technologies) and allowed to cure for 3 days prior to image acquisition (Little, et al. 2015; Abbaszadeh and Gavis 2016). Confocal microscopy was performed using a Leica STELLARIS 5 white light laser system photon counting mode for smFISH as described in detail (Niepielko, et al. 2018), whereas superresolution images were acquired using Leica LIGHTNING deconvolution with smooth rendering. Primordial germ cells were counted while rendered in 3D using Leica LAS X software.

### Single mRNA Identification and Homotypic Cluster Quantification

Identification and quantification of single transcript RNPs and homotypic clusters were performed using the custom MATLAB (Mathworks) program used in previous germ granule studies (Little, et al. 2011; Little, et al. 2015; Valentino, et al. 2022). In brief, we use a user defined polygon to mark the entire germ plasm within a z stack (∼5 µm,15 confocal slices). Next, quantification of the number of transcripts within a homotypic cluster was calculated by first setting an intensity threshold based on the average intensity of single transcript RNPs found outside of the posterior germ plasm and normalizing the intensity of germ plasm RNPs to the average intensity of single transcript RNPs (Little, et al. 2015). To determine whether homotypic clusters of *nos* and *pgc* reside within the same germ granule (referred to as colocalized), we calculate the frequency that two clusters’ centroids are found within a previously established distance threshold. Specifically, two previously established distance criteria were used: 1) the two homotypic clusters must be within a z distance of two slices for confocal images, and 2) they must be within a distance cutoff of 200 nm in *x*–*y*, which is a conservative distance based on the average size of *Drosophila* germ granules (Illmensee and Mahowald 1974; Amikura, et al. 2001; Little, et al. 2015; Eagle, et al. 2018; Niepielko, et al. 2018). Thus, the colocalization analysis does not refer to the intensities or physical overlap between different clusters; rather, it represents the fraction of clusters that are within the same germ granule based on distance. Following the identification and locations of *nos* and *pgc* clusters, the data were used to create the Granule Census as previously described in detail (Niepielko, et al. 2018; Valentino, et al. 2022). All confocal images shown in the figures were filtered by a balanced circular difference-of-Gaussian with a center radius size of 1.2 pixels and surround size of 2.2 pixels (Little, et al. 2015). To determine that single transcript RNPs diffuse through the bulk cytoplasm in non-*D. mel* species, each species’ smFISH experiments were first imaged under identical experimental and imaging conditions as *D. mel* samples, including using the same number of probes and dyes within a *nos* or *pgc* probe set. Next, single transcript thresholds from *D. mel* smFISH experiments were applied to *nos* or *pgc* RNPs found in the bulk cytoplasm's of non-*D. mel* species, producing comparable distributions between species where ∼85% of RNPs within the bulk cytoplasm were identified as singles (supplementary fig. S1*A* and *B*, Supplementary Material online). Some diffusing RNPs were calculated to have more than 1 transcript due to the random overlapping within the bulk cytoplasm that has been previously documented (Little, et al. 2015).

### Quantification of Transcript Levels

For all species, females were fed yeast for 24 h and stage 13/14 oocytes were dissected in cold PBS and isolated from the ovaries. Stage 13/14 oocytes were used since nurse cell dumping is completed at stage 12 (Spradling 1993), and therefore, all possible germ plasm transcripts will be located in the bulk cytoplasm pool or in germ granules within the germ plasm. Furthermore, expression levels from isolated stage 13/14 oocytes are consistent with input values for the computational model (Valentino, et al. 2022). Oocytes were homogenized, and RNA was extracted using RNeasy kit (Qiagen, cat #: 74104) using the provided protocol. Following RNA extraction, the QuantiTect Reverse Transcription Kit (Qiagen, cat #: 205311) was used to synthesize cDNA. For all species, qPCR experiments were performed using TaqMan Gene Expression Assays (ThermoFisher). In *D. mel*, *nos* (assay ID: Dm02134535_g1), *rpl7* (assay ID: Dm01817653_g1), and *osk* (assay ID: Dm02134538_g1) and a custom assay that was previously designed for *pgc* were used (Valentino, et al. 2022). All assays were carried out using TaqMan assay master mix (cat #: 4369514) using the included standard TaqMan protocol which was performed with a BIO-RAD CFX96 Real-Time System. Three technical replicates for a minimum of three biological replicates were performed for all qPCR experiments. Each biological replicate included >15 stage 13/14 oocytes that were collected from multiple females. For non-*D. mel* species, the custom TaqMan probe design tool (ThermoFisher) was used to design custom assays for *nos* and *pgc* using the same sequences as described for smFISH probe designs. For *rpl7* sequences, UniProt entry numbers B4M9C2 and Q29NI0 were used for *D. vir* and *D. pse*. For *osk* sequences, UniProt entry numbers B4LXK5 and A0A6I8URE4 were used for *D. vir* and *D. pse.* For *D. neb*, *rpl7* and *osk* sequences were determined using the *D. neb* annotated long-read sequenced genome (Sottolano, et al. 2022). For all expression assays, the presented fold gene expression levels are displayed as values relative to *D. mel* and were calculated using the 2(-Delta Delta C(T)) method (Livak and Schmittgen 2001) using *rpl7* as an internal control (Valentino, et al. 2022).

### Graphing and Statistical Analyses

All Granule Censuses and heatmaps were created using MATLAB (Mathworks) as previously described (Niepielko, et al. 2018). GCTAnalysis scores were calculated by first subtracting two Granule Censuses to create a new matrix that represents the difference between a census pair. Next, we calculated the magnitude of the new matrix using the norm function in MATLAB (Mathworks), resulting in a value that denotes the magnitude of change between a set of Granule Censuses. Additional graphs were generated using R statistical programming (R Core Team 2021), RStudio (RStudio Team 2020), and the ggplot2 package (Wickham 2016). The Venn diagram was created using the eulerr R package (Larsson 2022), undirected network graphs were created using the igraph R package (Csardi and Nepusz 2006), and the density plots and their percent overlap values were calculated using the overlapping R package (Pastore 2018; Pastore and Calcagni 2019). One-way ANOVA with Dunnett's post hoc tests were performed using the DescTools R package (Signorell 2022) to determine statistical significance when comparing average values from each species to *D. mel*, including comparing average sizes of bulk cytoplasm *nos* or *pgc* RNPs from three biological replicates (see bulk cytoplasm *P* values in fig. 2).

### Fly Strains and Cloning

The *y^1^, w^67c23^* strain, Bloomington Drosophila Stock Center (BDSC) 6599, was used as *D. mel*. To label the germ granule protein ensemble in figure 1, the *osk-gfp* transgene (fTRG_1394), a gift from H. Jambor, was used (Sarov, et al. 2016). *D. virilis*, *D. pseudoobscura*, and *D. nebulosa*, gifts from the Yakoby Lab (Niepielko, et al. 2011; Niepielko, et al. 2014), were used in this study. The *D. mel ^neb nos^*^3′ UTR^ and *D. mel ^pse nos^*^3′ UTR^ transgenes were created by first isolating species-specific *nos* 3′ UTR from genomic DNA using PCR with primers with EcoRI and XhoI cut sites engineered at the ends of the forward and reverse primers and amplified with Phusion DNA Polymerase (NEB). Primers were designed based on the *D. pse nos* sequence used to design the smFISH probe, and the *nos* sequence for *D. neb* was identified from a long-read sequenced genome (Sottolano, et al. 2022). Next, we removed the *nos* 3′ UTR from a 4.3 kb *D. mel* genomic nos rescue fragment, a gift from the Gavis Lab (Gavis and Lehmann 1992), using EcoRI and XhoI (NEB) and ligated species-specific 3′ UTR with T4 DNA ligase (NEB). Each of the full-length chimeric *nos* rescue fragments were cloned into pattB vectors using NotI (NEB) cut sites and inserted into the attP40 landing site by phiC31-mediated recombination (Bischof, et al. 2013). Injection services were provided by Rainbow Transgenics. The chimeric *nos* transgenes were introduced into *nos^BNX^* homozygous females to create the *D. mel ^neb nos^*^3′ UTR^ and *D. mel ^pse nos^*^3′ UTR^ fly lines that only express chimeric *nos* mRNA, as similarly done previously (Valentino, et al. 2022). All fly stocks, including *non-D. mel* species, were maintained at 23 °C on standard cornmeal food (Bloomington Formulation, Genesee Scientific Cat #: 66-112). To validate chimeric sequences, constructs were sequenced by Eton Biosciences with a *nos* primer that binds near the 3′ end of *nos* coding region, GCGATCAAGGCGGAATCGTTCC.

### Computational Modeling and Sequence Analysis

Computational modeling was carried out as previously described (Valentino, et al. 2022) by adjusting the model to recapitulate each species’ biological data. Clustering factor (cf) values were determined as previously described (Valentino, et al. 2022). In summary, qPCR data were first applied to the model's parameters to recreate known biological conditions. Next, homotypic cluster formation was modeled with different clustering values while maintaining the known biological conditions. Next, standard curves were generated based on plotting clustering factor values against the average cluster sizes that they produced. For *pgc* in *D. pse*, we used a standard curve generated at stage 13 since the largest *pgc* cluster sizes were detected at this stage. Biological averages were then fitted to the standard curve to determine the clustering factor value for the biological data. Given the decrease in *pgc* cluster sizes from stage 13 to early embryo in *D. pse* biological data, we adjusted the *D. pse* model to capture this phenomenon by incorporating a loss function starting at stage 14. Since the model is based on calculating a granule's ability to gain and/or lose transcripts, we developed a loss function that increases a granule's probability to lose a *pgc* transcript by multiplying the size of a given *pgc* cluster by 0.009 at stage 14 that increases to 0.04 in the early embryo. Estimates of evolutionary divergence between germ plasm 3′ UTR sequences was determined using the maximum composite likelihood model (Tamura, et al. 2004), based on the ClustalW alignment created by the 3′ UTR sequences. All ambiguous positions were removed for each sequence pair (pairwise deletion option), and there were a total of 1,006 positions in the final data set for the *nos* 3′ UTR, 477 positions for the *pgc* 3′ UTR, 876 positions for the *cycB* 3′ UTR, and 598 for the *gcl* 3′ UTR. Analyses were conducted in MEGA X (Kumar, et al. 2018). 3′ UTR sequences were determined using UniProt entry A0A6I8UT44 for *D. pse* and B4LIV1 for *D. vir*. As for *cycB*, UniProt entry A0A6I8UW25 was used for *D. pse* and B4MCQ2 was used for *D. vir*. For *D. neb*, *cycB* and *gcl* sequences were determined using the *D. neb* annotated long-read sequenced genome (Sottolano, et al. 2022). Estimates of evolutionary divergence between Osk, Vas, and Tud amino acid sequences were conducted using the Poisson correction model (Zuckerkandl and Pauling 1965). Each analysis involved four amino acid sequences. All ambiguous positions were removed for each sequence pair (pairwise deletion option). There was a total of 663 positions in the final data set for Osk, 2,671 positions for Tud, and 671 positions for Vas. Evolutionary analyses were conducted in MEGA X (Kumar, et al. 2018).

## Supplementary Material

msad174_Supplementary_Data

## Data Availability

The data underlying this article will be shared on reasonable request to the corresponding author.
