# Supplementary material for: Germ Granule Evolution Provides Mechanistic Insight into Drosophila Germline Development
Source: Mol Biol Evol. 2023 Aug 1;40(8):msad174. doi: 10.1093/molbev/msad174 (PMC10414811; doi:10.1093/molbev/msad174)
Supplement: msad174_Supplementary_Data [file msad174_supplementary_data.zip › Doyle_et_al_2023_Supplement.pdf]

Supplement Figure 1:

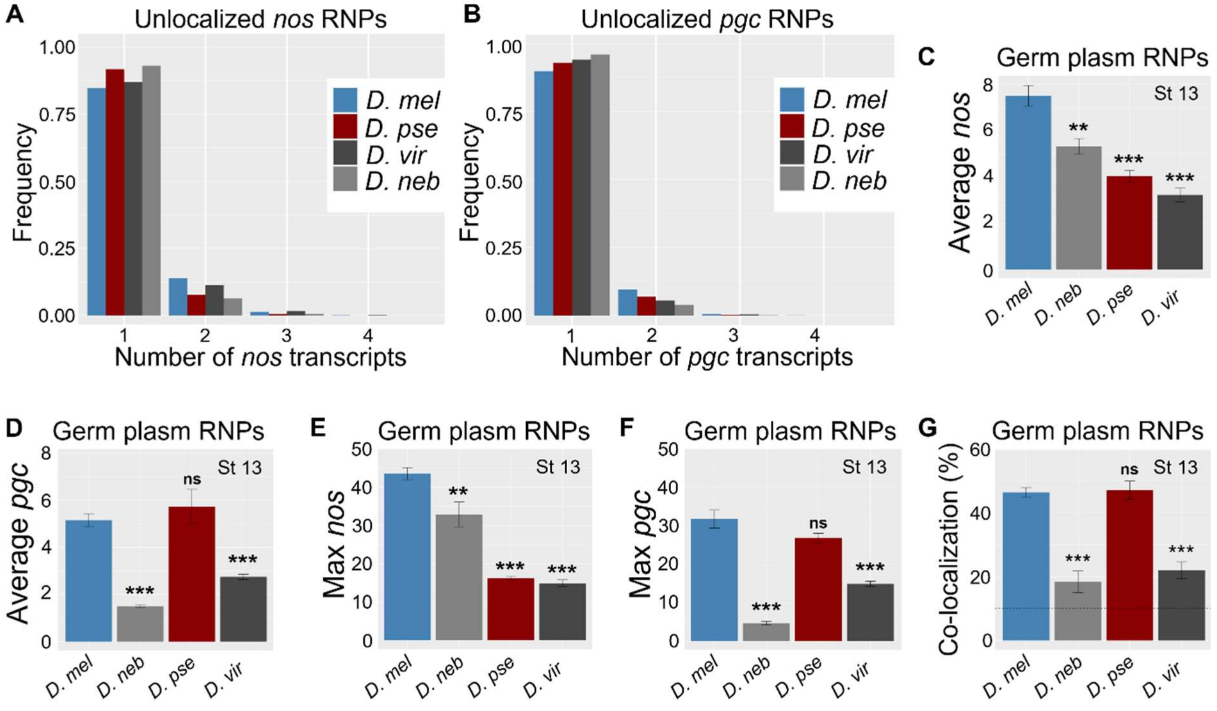

Supplement Figure 1: Number of *nos* and *pgc* transcripts in RNPs from the bulk oocyte cytoplasm and germ plasm. A) Distribution of the number of *nos* transcripts found in unlocalized RNPs in the bulk oocyte cytoplasm in all species analyzed n > 1,700 RNPs per species. **B)** Distribution of the number of *pgc* transcripts found in unlocalized RNPs in the bulk oocyte cytoplasm in each species tested n >1,900 RNPs per species. **C)** Average number of *nos* transcripts found in *nos* containing germ plasm RNPs found in each species in stage 13 oocytes. **D)** Average number of *pgc* transcripts found in *pgc* containing germ plasm RNPs found in each species in stage 13 oocytes. **E)** Largest number of transcripts (max) observed in *nos* containing germ plasm RNPs for each species in stage 13 oocytes. **F)** Max number of *pgc* transcripts found in *pgc* containing germ plasm RNPs for each species in stage 13 oocytes. **G)** Co-localization rate between *nos* and *pgc* containing RNPs in the germ plasm for each species in stage 13 oocytes. For C-G n > 5,000 germ plasm RNPs for each mRNA type. All error bars represent S.E.M. based on data collected from a minimum of 3 stage 13 oocytes. The dotted horizontal line represents the expected rate for random co-localization. P- value is represented by not significant (ns) > 0.05, ** < 0.01, and *** < 0.001 when compared to *D. mel*.

Supplement Figure 2:

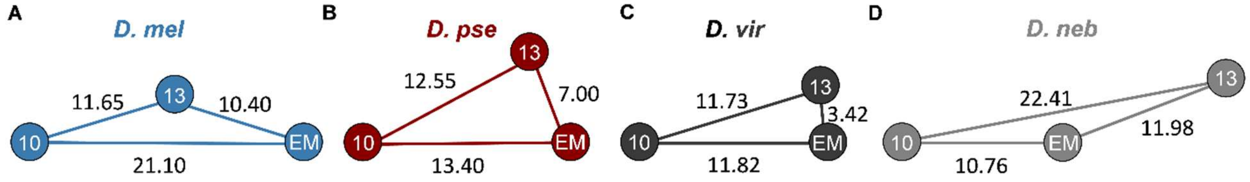

Supplement Figure 2: Representation of germ granule assembly dynamics using GCTAnalysis scores. A-D) Visual representation of germ granule formation dynamics where each edge represents a GCTAnalysis score that was calculated between each developmental stage, represented as nodes.

Supplement Figure 3:

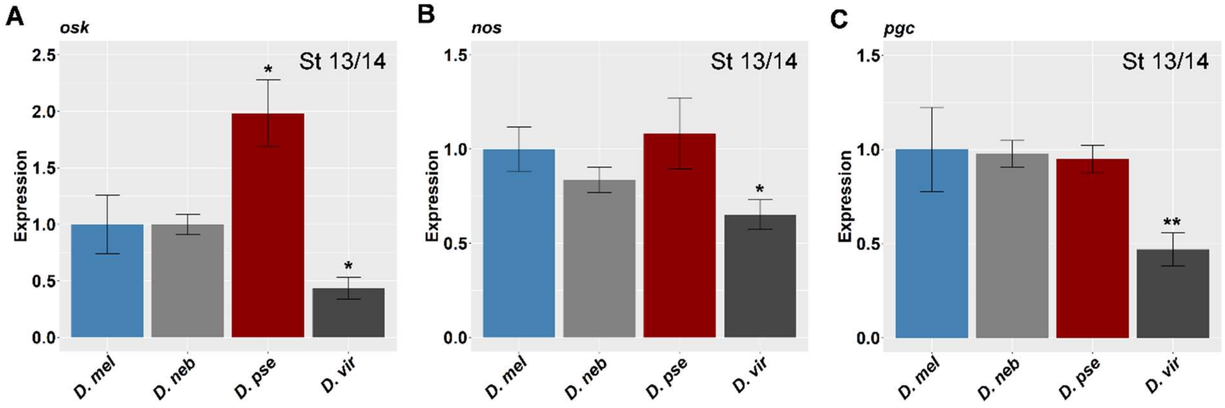

Supplement Figure 3: Relative mRNA expression levels of *osk, nos*, and *pgc* in different *Drosophila* species. A-C) Relative mRNA expression levels for **A)** *osk*, **B)** *nos*, and **C)** *pgc*. All expression levels are relative to *D. mel* and were measured from stage 13/14 oocytes. Error bars represent S.E.M. from a minimum of 3 biological replicates.

Supplement Figure 4:

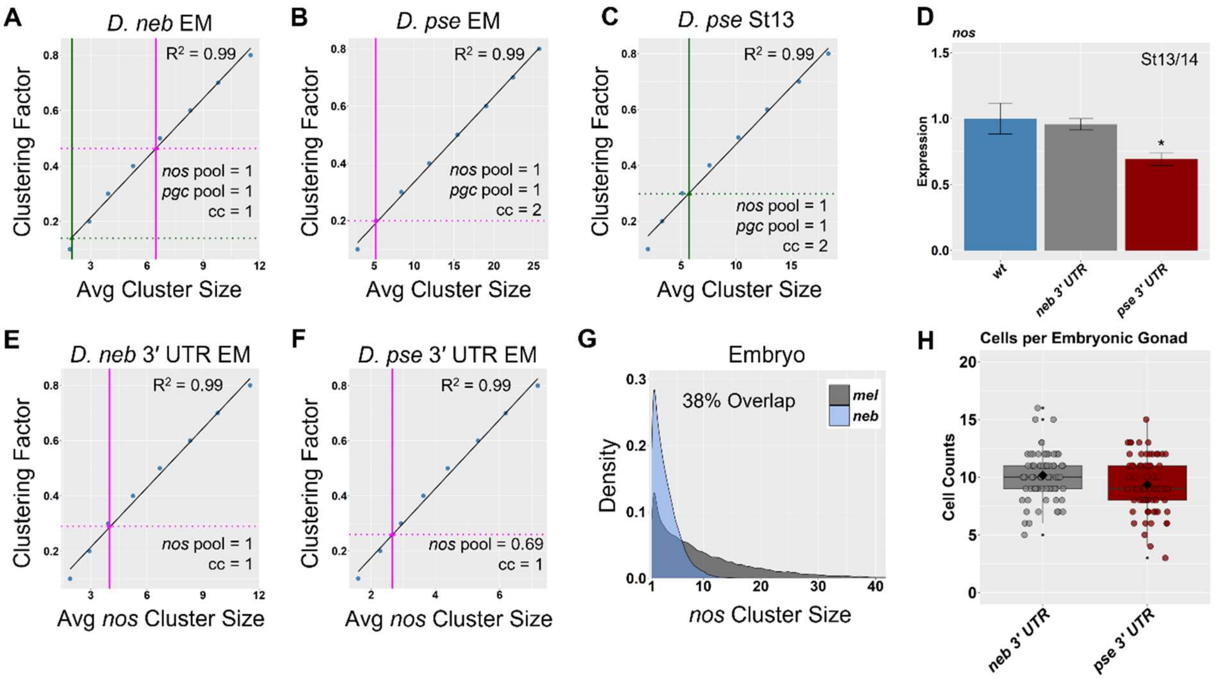

Supplement Figure 4: Calculating the clustering factor parameter, relative *nos* expression levels from recombinant flies, *nos* cluster size density plots, and gonad cell counts. A-C) Standard curves produced by the computational model using the biological determined parameters for *nos* expression (*nos* pool), *pgc* expression (*pgc* pool), and *osk* expression which represents carrying capacity (cc). The standard curves (solid black line) are produced using the average cluster size (x-axis) that is produced for a given clustering factor (y-axis). Fitting the biologically determined average cluster size for *nos* (vertical magenta line) and average cluster size for *pgc* (vertical green line) reveals the clustering factor for *nos* (broken horizontal magenta line) and for *pgc* (broken horizontal green line) for **A)** *D. neb* embryo, **B)** *D. pse* embryo, and **C)** *D. pse* St13. **D)** Relative *nos* expression levels from recombinant *D. mel* flies that contain the *D. neb* or the *D. pse nos* 3′ UTR. Expression levels are relative to wild type *D. mel nos* levels and were measured from stage 13/14 oocytes. Error bars represent S.E.M from a minimum of 3 biological replicates. **E-F)** Standard curves produced by the computational model using the biological determined parameters for *nos* expression (*nos* pool) and *osk* expression which represents carrying capacity (cc). The standard curves (solid black line) are produced using the average *nos* cluster size (x-axis) that is produced for a given clustering factor (y-axis). Fitting the biologically determined average cluster size for *nos* (vertical magenta line) reveals the clustering factor for *nos* (broken horizontal magenta line) for **E)** *D. neb nos* 3′ UTR in *D. mel* and **F)** *D. pse nos* 3′ UTR in *D. mel*. **G)** Density plots for *nos* cluster sizes in the embryo for wild-type *D. mel* (gray) and *D. mel* that contains the *nos* 3′ UTR from *D. neb* (blue), plots were generated using >17,500 *nos* clusters for each genotype from a minimum of 4 germ plasms. **H)** The number of Vasa positive cells within an embryonic gonad in *D. mel* $^{neb\ nos\ 3'\ UTR}$ and *D. mel* $^{pse\ nos\ 3'\ UTR}$, black diamond represents the average.

Supplement Figure 5:

A

The number of amino acid substitutions per site between sequences

	D. mel OSK	*D. pse* OSK	*D. vir* OSK	*D. neb* OSK
D. mel OSK				
D. pse OSK	0.558			
D. vir OSK	0.556	0.530		
D. neb OSK	0.671	0.653	0.459	

	D. mel TUD	*D. pse* TUD	*D. vir* TUD	*D. neb* TUD
D. mel TUD				
D. pse TUD	0.363			
D. vir TUD	0.441	0.436		
D. neb TUD	0.454	0.451	0.448	

	D. mel VAS	*D. pse* VAS	*D. vir* VAS	*D. neb* VAS
D. mel VAS				
D. pse VAS	0.388			
D. vir VAS	0.376	0.324		
D. neb VAS	0.409	0.346	0.270	

B

The number of base substitutions per site between sequences

	mel cycB 3′ UTR	*pse cycB* 3′ UTR	*vir cycB* 3′ UTR	*neb cycB* 3′ UTR
mel cycB 3′ UTR				
pse cycB 3′ UTR	0.541			
vir cycB 3′ UTR	1.033	0.930		
neb cycB 3′ UTR	1.326	1.327	1.042	

	mel gcl 3′ UTR	*pse gcl* 3′ UTR	*vir gcl* 3′ UTR	*neb gcl* 3′ UTR
mel gcl 3′ UTR				
pse gcl 3′ UTR	0.486			
vir gcl 3′ UTR	0.873	0.939		
neb gcl 3′ UTR	1.256	1.383	0.749	

	mel pgc 3′ UTR	*pse pgc* 3′ UTR	*vir pgc* 3′ UTR	*neb pgc* 3′ UTR
mel pgc 3′ UTR				
pse pgc 3′ UTR	0.539			
vir pgc 3′ UTR	1.057	0.986		
neb pgc 3′ UTR	1.247	1.272	1.423	

Supplement Figure 5: Evolutionary divergence of gem granule proteins and 3′ UTRs of germ granule mRNAs. A) The number of amino acid substitutions per site in Oskar, Tudor, and Vasa between *D. mel*, *D. pse*, *D. vir*, and *D. neb*. **B)** The number of base substitutions per site in the 3′ UTRs of *Cyclin B* (*cycB*), *germ cell-less* (*gcl*), and *polar granule component* (*pgc*) in *D. mel*, *D. pse*, *D. vir*, and *D. neb.*

Movies 1-3: Computational modeling representing the development of germ granule diversity in different *Drosophila* species. Movie 1) *D. vir*, **Movie 2)** *D. pse*, and **Movie 3)** *D. neb*. The horizontal magenta line represents the average *nos* cluster size while the green vertical line represents average cluster size of *pgc.* Y-axis represents *pgc* cluster size while the X-axis represent *nos* cluster size. The heatmap represents the number of germ granules with a particular mRNA composition. Time scale is 19 hours with snapshots at every 15 minutes.
