# Supplementary figures and images for: Germ Granule Evolution Provides Mechanistic Insight into Drosophila Germline Development
Source: Mol Biol Evol. 2023 Aug 1;40(8):msad174. doi: 10.1093/molbev/msad174 (PMC10414811; doi:10.1093/molbev/msad174)

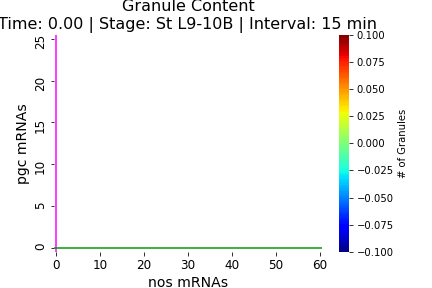

Supplement: msad174_Supplementary_Data [file msad174_supplementary_data.zip › movie_2_pse_animated_census.gif]

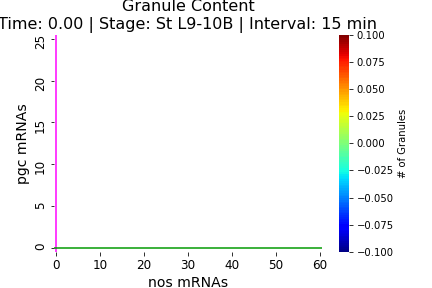

Supplement: msad174_Supplementary_Data [file msad174_supplementary_data.zip › movie_3_neb_animated_census.gif]

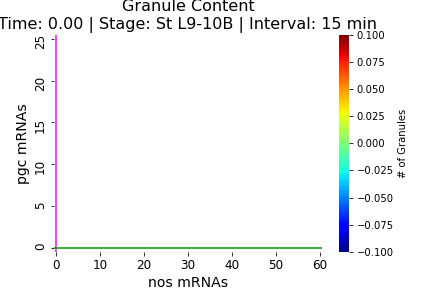

Supplement: msad174_Supplementary_Data [file msad174_supplementary_data.zip › movie_1_virilis_animated_census.gif]
